# Microplastic and trace element contamination in coastal agricultural soils of southern India: a comparative risk assessment of mulched and unmulched fields

**DOI:** 10.1007/s10653-025-02746-9

**Published:** 2025-09-14

**Authors:** Chattanchal Ashwathi, Anish Kumar Warrier, Monalisha Murmu, Udita Priyadarsini, Santhosh Prabhu

**Affiliations:** 1 Department of Civil Engineering, Manipal Institute of Technology, Manipal Academy of Higher Education, Manipal, Udupi, Karnataka 576104 India; 2 Department of Civil Engineering, Centre for Climate Studies, Manipal Institute of Technology, Manipal Academy of Higher Education, Manipal, Udupi, Karnataka 576104 India

**Keywords:** Emerging contaminants, Food security, Soil quality, Agriculture, Southwest India

## Abstract

This study investigates the occurrence and ecological risks of microplastics (MPs) and trace elements in coastal agricultural soils from Karnataka, southern India. Surface and subsurface soils from mulched (watermelon) and unmulched (rice) fields were analyzed for MP abundance, morphology, polymer composition, and trace elements. Microplastics were identified using Attenuated Total Reflectance–Fourier Transform Infrared Spectroscopy (ATR-FTIR), and metals were quantified via Inductively Coupled Plasma–Optical Emission Spectroscopy (ICP-OES). Scanning Electron Microscopy with Energy Dispersive X-ray Spectroscopy (SEM–EDS) confirmed elemental associations on MP surfaces. Risk was assessed using the Polymer Hazard Index (PHI), Pollution Load Index (PLI), Geoaccumulation Index (I_geo_), and Potential Ecological Risk Index (PERI). Results showed higher MP concentrations in surface soils and mulched fields, with polyethylene (PE) as the dominant polymer. Most MPs were 0.1–0.3 mm (49–50%) or 0.3–1 mm (40–47%) in size. Films were the most common type (61%), followed by fibres and fragments, with fibres dominating deeper layers. Transparent MPs were the most abundant, followed by blue, white, and black. The Polymer Hazard Index (PHI) indicated high risk. The Coefficient of Microplastic Impact (CMPI) showed a moderate impact, while the Pollution Load Index (PLI) values for MPs indicated low to moderate pollution, with particularly elevated levels in mulched soils. The Potential Ecological Risk Index (PERI) classified risk levels as low to moderate, with higher values also observed in mulched fields. Among trace elements, the concentration followed the order: Fe > Zn > Mn > Cr > Pb. While PLI and I_geo_ suggested low to moderate contamination, PERI indicated low ecological risk. These findings highlight how plastic mulching increases MP accumulation and alters metal dynamics in soil. The co-occurrence of MPs and metals raises concerns for soil health and food safety. Sustainable land management and regular monitoring are critical to mitigate long-term risks.

**Graphical abstract:**

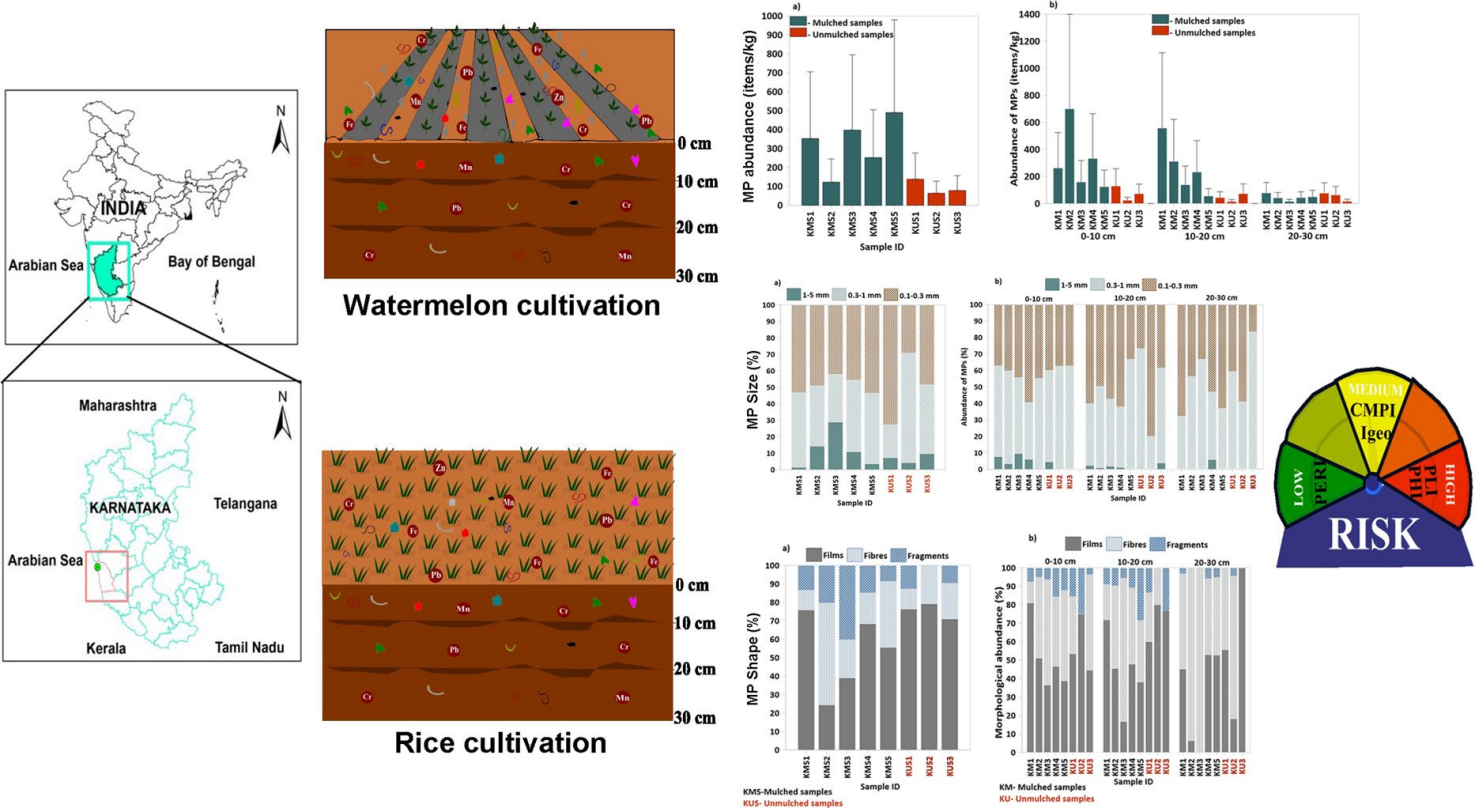

**Supplementary Information:**

The online version contains supplementary material available at 10.1007/s10653-025-02746-9.

## Introduction

Microplastics (MPs), defined as synthetic polymer particles ranging from 0.1 to 5 mm in size (Thompson et al., 2004), are increasingly recognized as a persistent and emerging contaminant in terrestrial ecosystems. Initially studied in marine environments, MPs are now being reported with growing concern in agricultural soils, where they may adversely affect soil structure, microbial communities, nutrient cycling, and overall crop productivity (Koyuncuoğlu & Erden, 2021; Lwanga et al., 2022; Padervand et al., 2020). MPs enter terrestrial systems through diverse sources, including wastewater irrigation, compost, biosolids, atmospheric deposition, and plasticulture practices such as mulching (Guo et al., 2025; Henseler et al., 2022; Khalid et al., 2023).

Plastic mulching, widely adopted to conserve soil moisture and improve yields, has emerged as a significant contributor to MP pollution in soils (Chen et al., 2015; Qi et al., 2020). Polyethylene-based mulch films degrade under photolytic and mechanical stress, fragmenting into MPs that accumulate in surface layers and potentially migrate into deeper soil horizons (Huang et al., 2020; Li et al., 2023; Steinmetz et al., 2016). Even in unmulched fields, MPs may infiltrate through indirect pathways such as surface runoff and wind transport (Kim et al., 2021). Understanding the vertical distribution and morphological characteristics of MPs in mulched versus unmulched systems is therefore critical for assessing their environmental impacts (Jamil et al., 2024). Alongside MPs, agricultural soils are increasingly contaminated by trace elements, primarily from agrochemicals, irrigation water, and atmospheric inputs (Yildiz & Ozkul, 2024). These metals impair soil fertility and pose significant health risks through food chain transfer (Shetty et al., 2025; Wuana & Okieimen, 2011). For example, lead (Pb) concentrations as low as 0.005 ppm have been shown to significantly inhibit root growth in crops such as lettuce and carrot (Nagajyoti et al., 2010).The persistence and toxicity of trace elements underscore the need for regular monitoring and risk assessment in agricultural settings. Cadmium (Cd) levels as low as 0.5–1.0 mg/kg can adversely affect soil enzymatic activity and plant nutrient uptake, while Pb concentrations exceeding 50 mg/kg are known to reduce microbial diversity and compromise soil functionality (Nagajyoti et al., 2010; Kabata-Pendias & Pendias, 2011; Wuana & Okieimen, 2011).

An emerging concern is the interactive behavior of MPs and trace elements in soil. MPs can serve as vectors by adsorbing toxic metals, altering their mobility, bioavailability, and ecological effects (Kajal & Thakur, 2024). This interaction may enhance metal uptake by plants and impact soil microbial health, while also facilitating the vertical and lateral transport of contaminants through the soil profile (Yang et al., 2023). Despite increasing recognition of such interactions globally, systematic investigations in Indian agricultural contexts remain scarce. In India, studies assessing MP contamination in soils are still limited (Kumar & Sharma, 2020; Shivaswamy et al., 2023; Singh et al., 2024), and even fewer have addressed their co-occurrence with trace elements or their depth-wise variability under different land-use practices (Mahreen et al., 2025). This gap is particularly evident in intensively cultivated regions such as coastal Karnataka, where plastic mulch use is common. Although several studies have investigated surface-level MP contamination, none have simultaneously examined the vertical distribution patterns of both MPs and trace elements across different cultivation systems. This study addresses these knowledge gaps by evaluating the spatial distribution, morphology, polymer composition, and ecological risk of MPs and trace elements in agricultural soils of Kirimanjeshwara, Udupi district. Surface and subsurface soils were sampled from mulched (watermelon) and unmulched (rice) fields to assess the influence of land management practices on contaminant accumulation and interaction. This work supports the UN SDGs by linking mulching practices to reduced soil contamination from microplastics and heavy metals, thereby promoting sustainable agriculture (SDG 2, SDG 12) and protecting terrestrial ecosystems (SDG 15).

## Materials and methods

### Study area and sampling

**Fig. 1:**
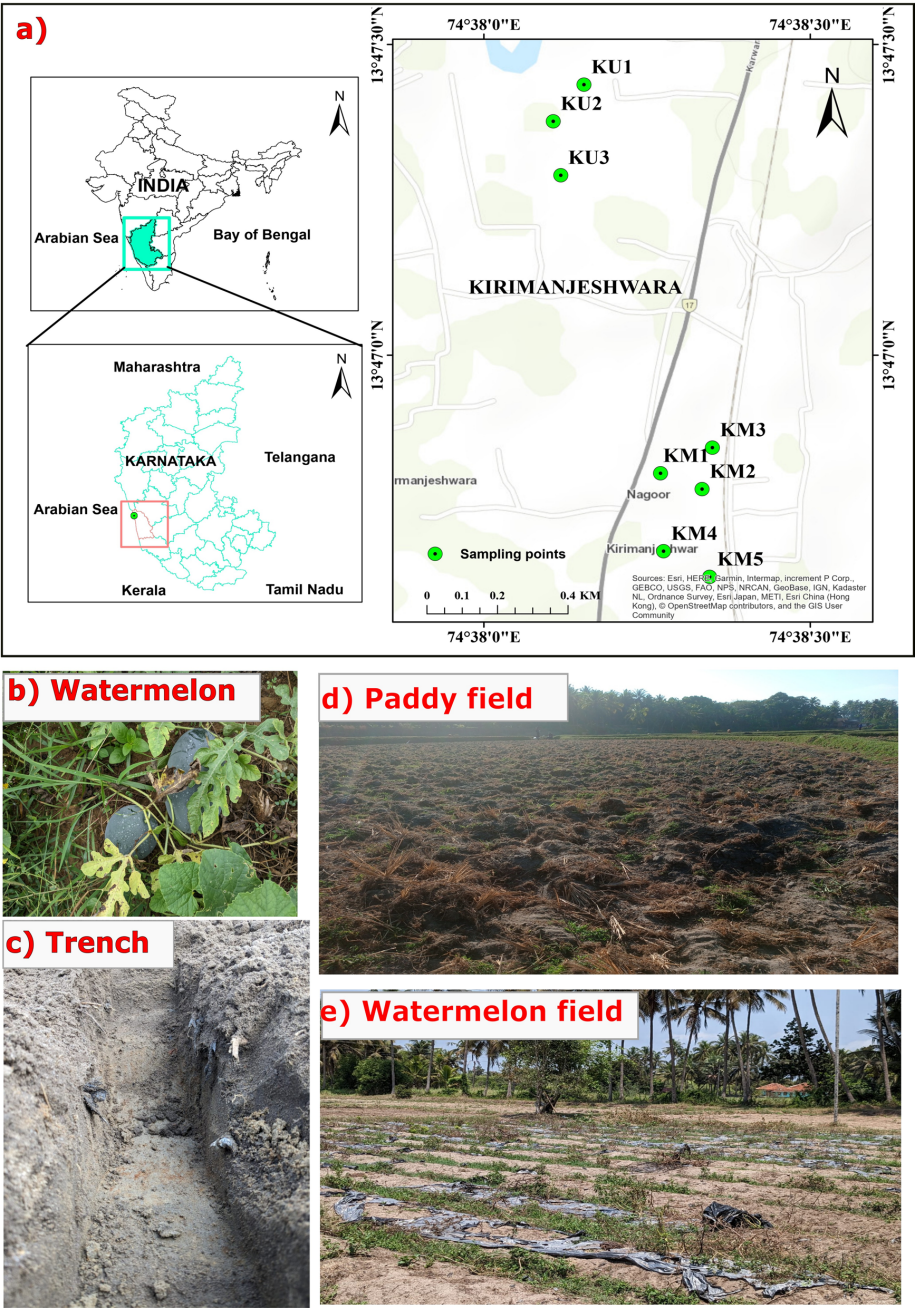
**a** Map showing the location of the study area in Kirimanjeshwara, Udupi district. Sample IDs ‘KM’ refer to mulched fields, and ‘KU’ refer to unmulched fields. **b** Watermelon cultivation in a mulched agricultural field. **c** Excavated trench for subsurface soil sampling. **d** View of an unmulched paddy field in Kirimanjeshwara. **e** View of a mulched watermelon field in Kirimanjeshwara

Kirimanjeshwara (Latitude: 13° 46′ 33.6″, Longitude: 74° 37′ 55.2″) is a small coastal village located along the Arabian Sea in Udupi district, Karnataka, India (Fig. 1a). The total geographic area of the village is 772.62 hectares (District Environmental Plan for Udupi District, Office of the Deputy Commissioner, 2021). Geologically, the area forms part of Peninsular India and is underlain by a Tertiary to Quaternary sedimentary cover composed of sands, silts, and clays, which rest atop the Precambrian Peninsular Gneissic Complex and fragments of the Dharwar Craton. The granitic-gneissic basement has undergone intense chemical weathering due to the region’s tropical monsoonal climate, resulting in the development of thick lateritic profiles. Coastal alluvium and lowlands are characterized by clay-rich deposits, while fine silica sands dominate the estuarine and beach environments (Radhakrishna & Vaidyanadhan, 2024). The region supports a mixed rural economy, with agriculture, fishing, and local handicrafts serving as the primary sources of livelihood. It experiences a tropical monsoon climate, receiving an average annual rainfall of approximately 4136.3 mm during the southwest monsoon (Sunilkumar et al., 2025). The mean annual temperature is 26.5 °C, with summer temperatures reaching up to 38 °C and winter temperatures typically ranging between 20 and 32 °C (Achari et al., 2023; Jayashree et al., 2023).

Soil sampling was conducted in May 2023 from agricultural fields subjected to different land management practices. A total of eight fields were selected, comprising five mulched fields cultivated with watermelon and three unmulched fields cultivated with paddy. From these sites, thirty-two composite soil samples, each weighing approximately 1 kg, were collected for the analysis of MP and trace elements. Of the total, eight were surface soil samples (0–10 cm), and twenty-four were subsurface samples collected from three distinct depth intervals: 0–10 cm, 10–20 cm, and 20–30 cm, with eight samples obtained from each interval. Subsamples from each depth were homogenized in the field to form representative composite samples prior to laboratory analysis.

Surface soil samples were collected by combining five subsamples taken from the four corners and the centre of each designated sampling plot. These subsamples were homogenized in the field to form a representative composite sample for each site. For subsurface sampling, three trenches were excavated within each field. At each trench location, soils were collected from the predefined depth intervals (0–10 cm, 10–20 cm, and 20–30 cm). Subsamples from the same depth across the three trenches were thoroughly mixed to generate a composite sample representative of each depth layer.

Following soil collection, samples designated for MP analysis were carefully packed in pre-cleaned aluminium containers to avoid external contamination, while those intended for trace elements analysis were stored in labelled plastic zip-lock bags. All samples were transported to the laboratory under controlled conditions and stored at 4 °C in a deep freezer until subsequent processing and analysis.

### Microplastic extraction and analysis

Each composite soil sample (400 g) was oven-dried at 50 °C until a constant weight was achieved, to eliminate moisture and establish the dry weight basis for all subsequent analyses. Dried samples were treated with sodium hexametaphosphate as a dispersing agent and agitated on a rotary shaker at 200 rpm for 1 h to ensure disaggregation of soil particles. Primary density separation was carried out by adding 300 mL of zinc chloride solution (ZnCl_2_; 933.3 g/L) to each sample. After overnight settling, the supernatant containing buoyant particles was collected. This step was repeated twice to maximize MP recovery. The recovered fraction was sieved using stainless steel meshes with apertures of 5 mm and 0.1 mm. Particles larger than 5 mm were classified as macroplastics and excluded, while those between 0.1 and 5 mm were retained for MP characterization (Amrutha & Warrier, 2020; Valsan et al., 2024a, 2024b).

The retained fraction was oven-dried at 50 °C and treated with 30 mL of 30% hydrogen peroxide (H_2_O_2_) to remove organic matter. A secondary density separation using ZnCl_2_ was conducted, followed by sieving through stainless steel meshes of 1 mm, 0.3 mm, and 0.1 mm. The final residues were transferred into clean Petri dishes using pre-filtered distilled water and dried at 50 °C. Samples were examined under a Nikon SMZ 745 T stereo-zoom microscope, and suspected MPs were confirmed using the hot needle test (Hidalgo-Ruz et al., 2012).

Polymer identification was performed on 100 particles (0.3–5 mm) using Attenuated Total Reflectance–Fourier Transform Infrared Spectroscopy (ATR-FTIR). Of these, 74 were 1–5 mm and 26 were 0.3–1 mm. The particles were selected to capture variability in shape, size, and sampling location. Although the number analyzed is limited relative to the total MP abundance, such subsampling is a widely accepted practice in MP research when analytical capacity is constrained (Valsan et al., 2024a, 2024b). The approach yields an indicative profile of the dominant polymer types present in the system. Spectra were acquired over 4000–400 cm^−1^ and analyzed using the Open Specy software; only those with a Pearson correlation coefficient above 0.8 were confirmed as MPs (Cowger et al., 2021). Selected MPs (> 1 mm) were further analyzed for surface morphology and elemental composition using a scanning electron microscope (SEM, EVO MA18) equipped with an energy-dispersive X-ray spectroscopy (EDS) detector (Oxford X-act). Samples were sputter-coated with gold prior to imaging to enhance conductivity (Valsan et al., 2023).

### Analysis of trace elements

Approximately 0.2 g of each finely powdered soil sample was accurately weighed and subjected to acid digestion on a hotplate using a mixture of 5 mL of 69% nitric acid (HNO₃), 3 mL of 35% hydrochloric acid (HCl), and 2 mL of 48% hydrofluoric acid (HF), following the procedure described by Joju et al. (2024). After complete digestion and evaporation to near dryness, the residues were diluted to a final volume of 50 mL using 0.2 N HNO₃. The digested solutions were then filtered through cellulose acetate syringe filters to remove residual particulates (Nishitha et al., 2022). Concentrations of trace elements, including manganese (Mn), chromium (Cr), zinc (Zn), lead (Pb), and iron (Fe), were quantified using a Thermo Scientific iCAP 7000 Series Inductively Coupled Plasma–Optical Emission Spectrometer (ICP-OES).

### Quality control and quality assurance

To minimize contamination, all solutions and distilled water used throughout the analysis were pre-filtered using a 0.1 mm stainless steel sieve. Background contamination was assessed using three field blanks and four laboratory blanks, in which a total of nine MP particles were detected. These background values were subtracted from the final dataset to ensure accurate quantification of MPs. This value was applied as a field-wide background correction and subtracted uniformly from all samples to account for potential contamination during processing and analysis. While this approach provides a conservative estimate, future studies may benefit from implementing sample-specific blank corrections to enhance analytical precision. A recovery experiment was conducted using five pre-treated soil samples spiked with a known quantity of MPs—comprising 20 fibres, 15 fragments, 15 films, and 15 foams per sample, totaling 325 particles. The average recovery rate was 98.15%, indicating high extraction efficiency and method reliability.

For trace elements analysis, analytical accuracy was verified using certified reference material (ERM-CC 141; loam soil). The relative standard deviation (RSD) for replicate measurements was maintained below 5%. Quality assurance and control (QA/QC) protocols included the routine analysis of duplicate samples and calibration check standards to ensure the precision and reproducibility of Inductively Coupled Plasma–Optical Emission Spectrometry (ICP-OES) results.

### Statistical analysis

Prior to statistical analysis, the distribution of MP abundance data was assessed using the Shapiro–Wilk test. As the data deviated from normality, a log transformation [log(x)] was applied to standardize the dataset. Normality was re-evaluated post-transformation, after which a parametric Welch’s two-sample t-test was employed to compare MP abundance across different groups. All statistical analyses were conducted using R software (version 4.4.2). Principal Component Analysis (PCA) was performed to explore multivariate patterns in MP and trace elements data. Prior to analysis, all variables were standardized using z-score normalization. PCA was executed on the correlation matrix using PAST software (version 5.2.2; Hammer & Harper, 2001). The suitability of the dataset for PCA was confirmed through Bartlett’s test of sphericity and the Kaiser–Meyer–Olkin (KMO) measure of sampling adequacy. Components with eigenvalues greater than 1 were retained for interpretation. To enhance interpretability, varimax rotation was applied, and the resulting component loadings and biplots were used to assess spatial patterns based on land use (mulched vs. unmulched) and soil depth. The sampling site map was generated using ArcGIS software (version 10.3), and all other graphical representations and plots were prepared using Grapher software (version 16.0).

### Risk assessment studies of MPs

With the increasing detection of MPs across diverse environmental matrices, there is a growing need to evaluate their potential ecological risks. Environmental risk assessment frameworks provide a structured means of estimating the ecological impacts of MPs by integrating their distribution data with polymer-specific toxicity metrics. Among the available approaches, the Polymer Hazard Index (PHI) has gained prominence as a quantitative tool for assessing the relative hazard of MPs based on their polymer composition.

The PHI is calculated by summing the products of the proportion of each polymer type (Pₙ) and its associated hazard score (Sₙ), as proposed by Lithner et al., (2011). The resulting value categorizes MPs into one of five hazard levels: Level I (low risk, 0–1), Level II (moderate risk, 1–10), Level III (high risk, 10–100), Level IV (very high risk, 100–1000), and Level V (extremely high risk, > 1000). This index has proven useful in highlighting the ecological threat posed by toxic polymers such as polyvinyl chloride (PVC), polystyrene (PS), and polyurethane (PU), particularly when compared to relatively less hazardous polymers like polyethylene (PE) and polypropylene (PP) (Wiesinger et al., 2021).In addition to the Polymer Hazard Index (PHI), the Coefficient of Microplastic Impact (CMPI) serves as a valuable environmental assessment tool by incorporating the morphological diversity of MPs found in soil samples. This index aids in understanding the relative contribution of different MP shapes, which can offer insights into their potential sources and transport mechanisms (Khaleel et al., 2023; Valsan et al., 2024b). The calculation of CMPI is performed using the following formula:

$$CMPI = Specific\;MP\;shape/\left( {Total\;MPs} \right)$$

The classification system proposed by Rangel-Buitrago et al., (2021) categorizes CMPI values into four distinct impact ranges: minimum (0.0001–0.1), average (0.11–0.5), maximum (0.51–0.8), and extreme (0.81–1). These categories help in assessing the severity of MP contamination based on particle morphology and abundance. When used in conjunction, the PHI and CMPI provide complementary insights—linking the chemical toxicity of polymer types (via PHI) with the morphological distribution and potential source pathways of MPs (via CMPI). Together, they offer a comprehensive evaluation of the ecological burden posed by MP pollution in soil environments.

To assess the overall MP contamination across the study sites, the Pollution Load Index (PLI) was adapted and calculated. Although originally developed for evaluating heavy metal pollution (Tomlinson et al., 1980), the index was modified to suit MP pollution by using the contamination factor of MPs instead of metal concentrations (Aydin et al., 2025; Hoshyari et al., 2023; Rana et al., 2023; Zhang et al., 2025). The contamination factor (CF) for each site was calculated as:

$$CFn = Cn/Bn$$

where C_n_ represents the MP abundance (items/kg) at each site, and B_n_ signifies the corresponding background value, assumed in this case to be the lowest MP abundance observed across all sites (Tomlinson et al., 1980). The Pollution Load Index PLI at each site is then computed as: $PLI = \surd CFn$. To evaluate cumulative pollution across a larger spatial unit (e.g., a zone), the zonal PLI was calculated as the geometric mean of CF values across all sampled sites within that zone:

$$PLI_{Zone} = n\sqrt {(CF_{1} \times CF_{2} \times CF_{3} \times \cdots \times CF_{{\text{n}}} )} $$

This modified approach enables a comparative assessment of MP contamination intensity across individual sites and broader zones. A PLI value equal to 1 indicates baseline or background levels of microplastic (MP) contamination, whereas a value greater than 1 suggests MP pollution. Conversely, a PLI value less than 1 implies low or insignificant pollution relative to the reference site (Kabir et al., 2021).The Potential Ecological Risk Index (PERI) for MPs was adapted from Hakanson’s (1980) method by incorporating MP abundance and polymer-specific toxicity. The Contamination Factor (CF) was calculated as the ratio of MP abundance at each site to the reference (minimum observed) abundance. To account for polymer-specific hazard, a toxicity coefficient (Tr) was calculated using the following equation

$$T_{r} = \mathop \sum \limits_{n = 1}^{n} (P_{n} /C_{i} \times S_{n} )$$

where T_r_ is the toxicity coefficient at a given site, P_n_ is the abundance of polymer type *n*, C_i_ is the total MP concentration at the site, and S_n_ is the hazard score of each polymer based on Lithner et al. (2011).

The Ecological Risk Factor (Er) for each site was then obtained by multiplying the contamination factor with the site-specific toxicity coefficient:

$$Er = CF \times T_{r}$$

The PERI was computed by summing all *Er* values across detected polymer types at each site. Risk levels were classified as follows (Ranjani et al., 2021): PERI < 150 (low risk), 150–300 (moderate risk), 300–600 (considerable risk), 600–1200 (high risk), and > 1200 (very high risk).

### Risk assessment studies of trace elements

A comprehensive understanding and assessment of the potential hazards associated with trace element contamination in soils are critical for effective environmental management and the protection of public health. This requires the use of robust risk assessment frameworks capable of identifying, characterizing, and quantifying the likelihood and severity of adverse effects resulting from exposure to trace elements in the soil environment.

Among the various assessment tools, the Pollution Load Index (PLI) is a popular tool for evaluating the overall level of trace element contamination in a given area. The PLI is calculated based on the contamination factors of individual metals and serves as an integrated measure of pollution status. It is interpreted as follows: PLI > 1 indicates the presence of pollution PLI = 1 suggested baseline contamination levels; and PLI < 1 denotes no significant pollution. The PLI is computed using the following equations:

$$PLI = n\sqrt {(CF_{1} \times CF_{2} \times CF_{3} \times \cdots \times CF_{{\text{n}}} )}$$

where n is the total number of trace elements examined, CF_n_ is the contamination factor, whose formula is $CFn = Cn/Bn$, where C_n_ represents the concentration of a specific metal, while B_n_ signifies its corresponding background value which is assumed to be the lowest concentration of each trace element in this case (Tomlinson et al., 1980).

Another quantitative measure for determining the level of trace element pollution in soils is the Geoaccumulation Index (I_geo_). It is calculated using the equation:

$$I_{geo} = log_{2} \left( {\frac{{C_{n} }}{{1.5 \times B_{n} }}} \right)$$

where C_n_ is the concentration of the nth metal and B_n_ which is assumed to be the lowest concentration of each trace element. The factor 1.5 is a matrix correction factor used to stabilize against background changes due to lithogenic conditions (Rivera et al., 2015).

Muller (1969) have classified I_geo_ into 7 classes as follows: uncontaminated to moderately contaminated (Class 0, I_geo_  < 1), moderately contaminated (Class 2, 1 <  I_geo_  < 2), moderately to heavily contaminated (Class 3, 2 <  I_geo_  < 3), heavily contaminated (Class 4, 3 <  I_geo_  < 4), heavily to extremely contaminated (Class 5, 4 <  I_geo_  < 5), and extremely contaminated (Class 6, I_geo_  > 5).

The Potential Ecological Risk Index (PERI is a frequently employed tool for evaluating ecological risk posed by trace elements in soils. It not only considers the concentration of pollutants but also their toxicity levels, providing a more comprehensive understanding of the ecological risks (Pradit et al., 2024). The PERI is calculated as follows:

$$PERI = \sum Ei$$

where $Ei = Ti \times Ci/Co$. Here, the soil PERI quantifies the combined ecological risk of multiple elements. It's the sum of individual element risk factors (Ei), where each Ei is the product of an element's toxicity (Ti) and its concentration relative to background levels (Ci/Co). According to Hakanson (1980), toxicity factors for trace elements are classified as follows: Cr = 2, Mn = 1, Pb = 5, and Zn = 1. The resulting PERI classifies ecological risk as: low (< 150), moderate (150–300), considerable (300–600), and high (> 600). This index provides a comprehensive measure of the potential adverse effects of trace element contamination on soil ecosystems.

## Results

### Microplastic abundance in agricultural soils

**Fig. 2:**
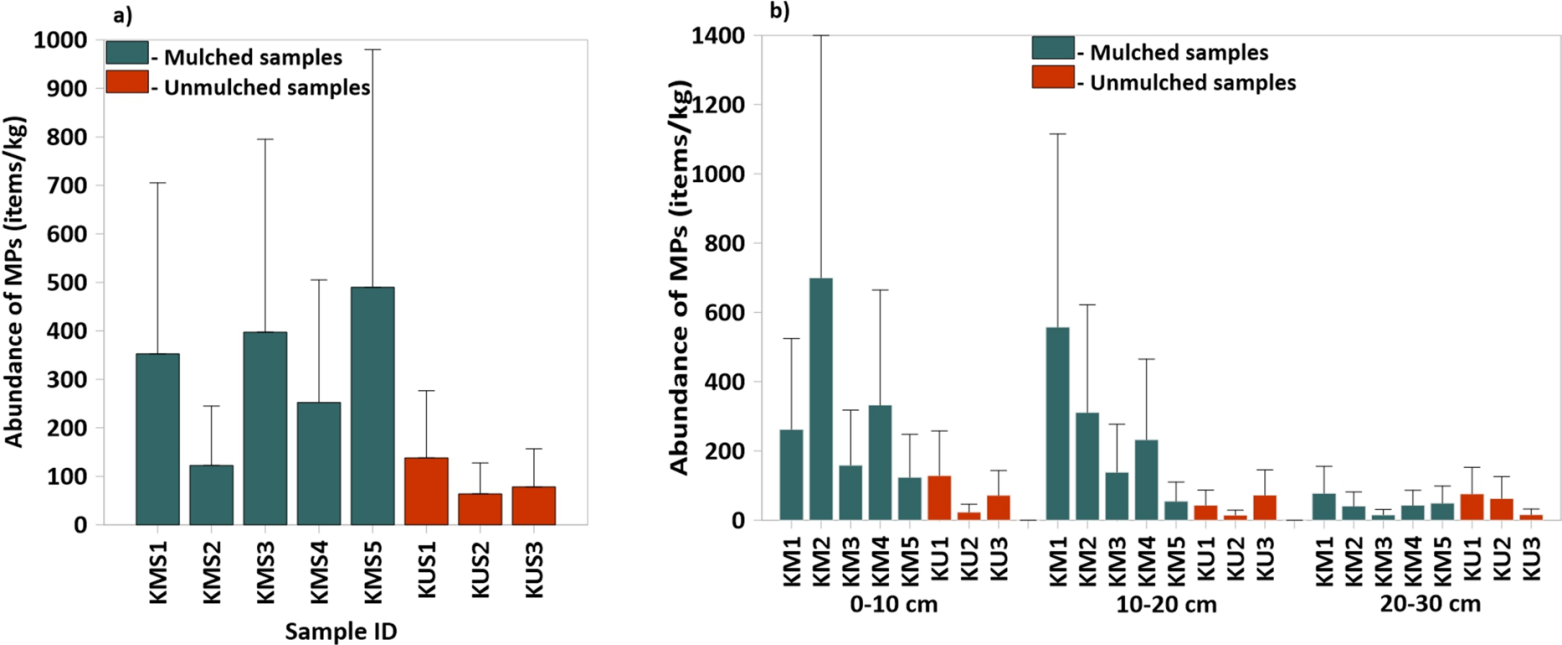
**a** Abundance of microplastics (MPs) in surface soil samples collected from agricultural fields in Kirimanjeshwara. **b** Vertical distribution of MP abundance across three soil depth intervals: 0–10 cm, 10–20 cm, and 20–30 cm. Error bars represent estimated counting uncertainty for each composite sample

The total abundance of MPs recovered from surface soil samples in Kirimanjeshwara was 1895.41 items/kg, with a mean (± standard deviation) value of 236.93 ± 160.97 items/kg (Fig. 2a). Statistical analysis indicated no significant difference in MP abundance across surface samples (*p* > 0.05). The MP abundance was assessed across three soil layers: 0–10 cm, 10–20 cm, and 20–30 cm—was recorded as 1802.6, 1426.5, and 382.98 items/kg, with respective mean values (± SD) of 225.32 ± 215.91, 178.32 ± 184.08, and 47.87 ± 24.14 items/kg. MP concentrations ranged from 23.38 to 700 items/kg in the top layer, 14.68 to 557.5 items/kg in the middle layer, and 15.63 to 77.98 items/kg in the deepest layer (Fig. 2b). While no significant differences were observed among the three depth layers overall (*p* > 0.05, n = 24), post hoc analysis showed that MP abundance at 20–30 cm was significantly lower than at 0–10 cm. (*p* < 0.05), suggesting a clear depth-dependent decline. These results indicate that MP concentrations decrease with increasing soil depth, with the highest accumulation occurring in surface layers.

Comparing between mulched and unmulched fields revealed a total MP abundance of 4715.7 items/kg in mulched soils, with a mean of 235.79 ± 186.53 items/kg, compared to 791.7 items/kg in unmulched soils, averaging 65.98 ± 37.63 items/kg (Supplementary Fig. S1). This represents an 83% higher MP abundance in mulched soils. Normality of abundance data was confirmed via the Shapiro–Wilk test (*p* > 0.05), allowing for the application of parametric tests without transformation. Welch’s two-sample t-test revealed a statistically significant difference in MP abundance between mulched and unmulched fields (t (21.58) = 3.84, *p* = 0.0009). The 95% confidence interval for the difference in means ranged from 77.89 to 261.72 items/kg, confirming that mulching is significantly associated with higher MP accumulation in agricultural soils.

### Abundance of trace elements

The overall concentration of trace elements in the soil samples followed the order: Fe > Zn > Mn > Cr > Pb. In surface soil samples, the average concentrations (mg/kg ± SD) were as follows: Fe (9195.79 ± 4162.47), Zn (284.98 ± 141.35), Mn (97.70 ± 18.96), and Cr (65.11 ± 22.33) (Supplementary Table S3). Notably, lead (Pb) was not detected in any of the surface samples, with measured concentrations consistently below detection limits. In subsurface samples, the mean concentrations were Fe (8803.57 ± 3070.83), Zn (315.45 ± 183.84), Mn (88.81 ± 24.48), Cr (69.27 ± 26.57), and Pb (54.23 ± 9.67) (Supplementary Table S4).

### Microplastic characteristics: shape, size, and colour

**Fig. 3:**
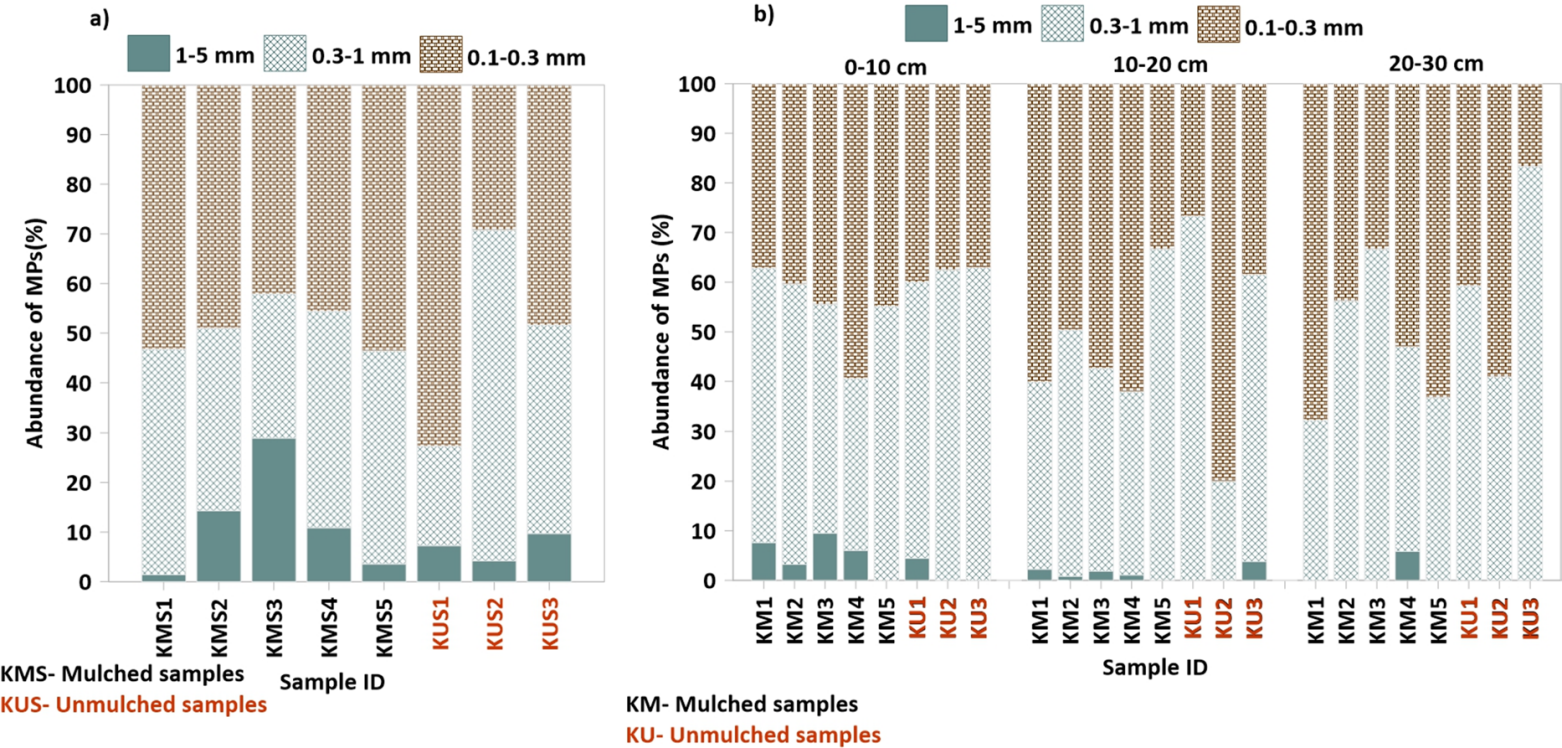
**a** Size distribution (%) of microplastics (MPs) in surface soil samples. **b** Size distribution (%) of MPs across three soil depth intervals: 0–10 cm, 10–20 cm, and 20–30 cm

The majority of MPs identified in surface soil samples belonged to the 0.1–0.3 mm size class, accounting for 49.21% of all MPs detected. This was followed by the 0.3–1 mm fraction (40.76%), while the largest size class (1–5 mm) comprised only 10.03% of the total (Fig. 3a). A similar pattern was observed in the 10–20 cm depth samples, where 0.1–0.3 MPs dominated (50.94%), followed by 0.3–1 mm (47.83%), and 1–5 mm (1.23%). In contrast, the 0–10 cm and 20–30 cm depth intervals showed a reversal in dominance between the smallest and medium size classes. At 0–10 cm depth, MPs in the 0.3–1 mm size range were most prevalent (53.55%), followed by 0.1–0.3 mm (42.6%), and 1–5 mm (3.85%). Similarly, in the 20–30 cm layer, 0.3–1 mm MPs constituted 52.09%, 0.1–0.3 mm accounted for 47.18%, and only 0.73% were in the 1–5 mm range (Fig. 3b).

When categorized by field type, mulched samples showed a predominance of MPs in the 0.1–0.3 mm range (49.65%), followed by 0.3–1 mm (45.48%) and 1–5 mm (4.87%). In contrast, unmulched samples exhibited a shift, with the 0.3–1 mm fraction being most dominant (53.68%), followed by 0.1–0.3 mm (43.87%) and 1–5 mm (2.45%) (Fig. 3).

**Fig. 4:**
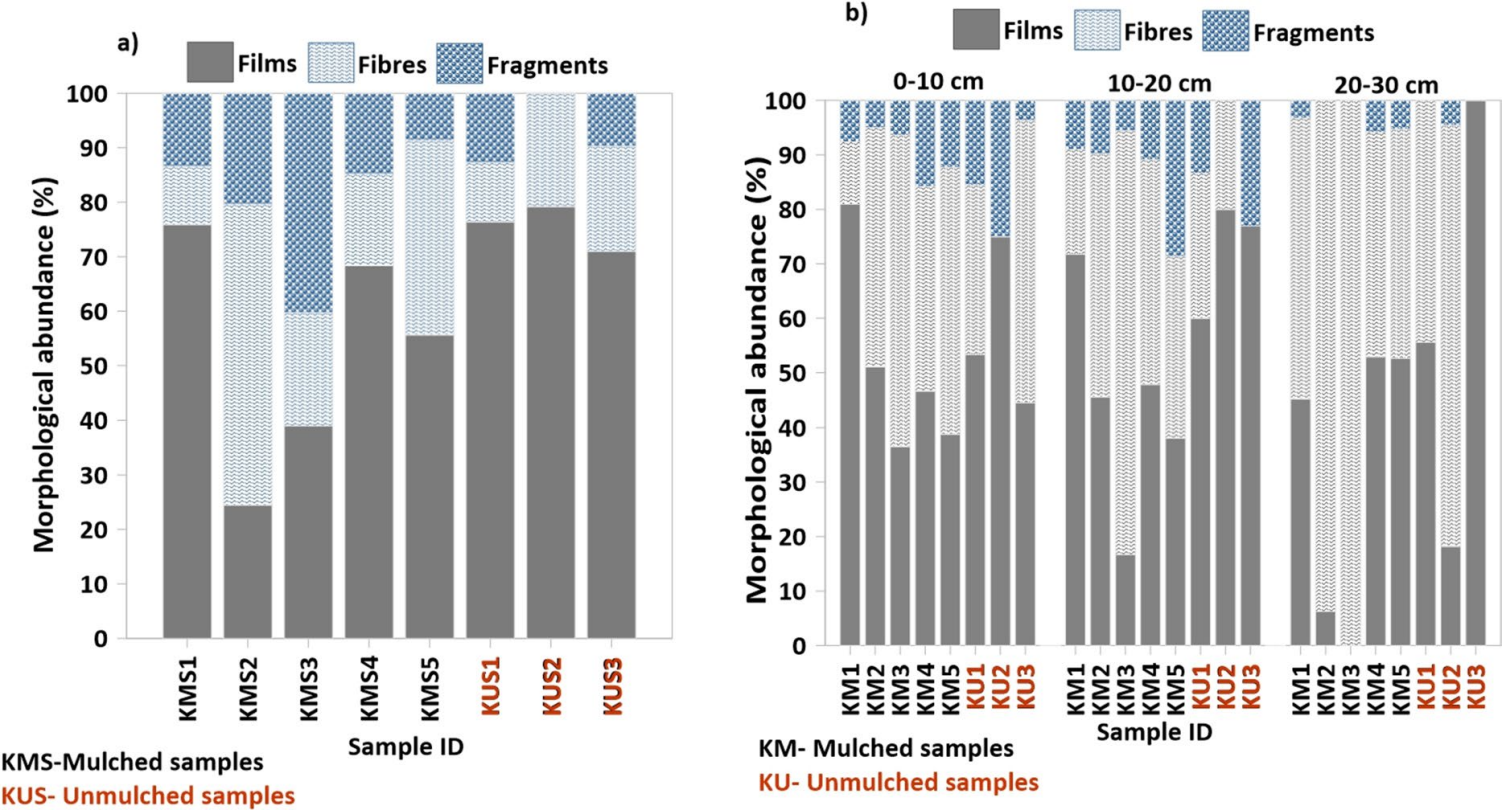
**a** Percentage distribution of microplastic (MP) morphological categories (films, fibres, and fragments) in surface soil samples. **b** Percentage distribution of MP categories across three soil depth intervals: 0–10 cm, 10–20 cm, and 20–30 cm

Microplastics identified across all soil samples belonged to three morphological categories: films, fibres, and fragments. Notably no foams or pellets were detected in any of the samples (Fig. 4; Supplementary Tables S1 and S2). Films were the most dominant MP type in the agricultural soils of Kirimanjeshwara, comprising 61.22% of all MPs in surface samples, followed by fibres (23.77%) and fragments (15.00%) (Fig. 4a). This trend was consistent across both mulched and unmulched fields.

A depth-wise analysis revealed variations in MP morphology. Within the 0–10 cm soil depth, films were found to be the predominant form (53.34%), followed by fibres (35.25%) and fragments (11.41%) (Fig. 4b). A similar pattern was observed at the 10–20 cm depth, with films constituting 54.60%, fibres 32.89%, and fragments 12.51%. However, at the 20–30 cm depth, fibres became the dominant type (56.30%), followed by films (41.34%) and fragments (2.36%).

**Fig. 5:**
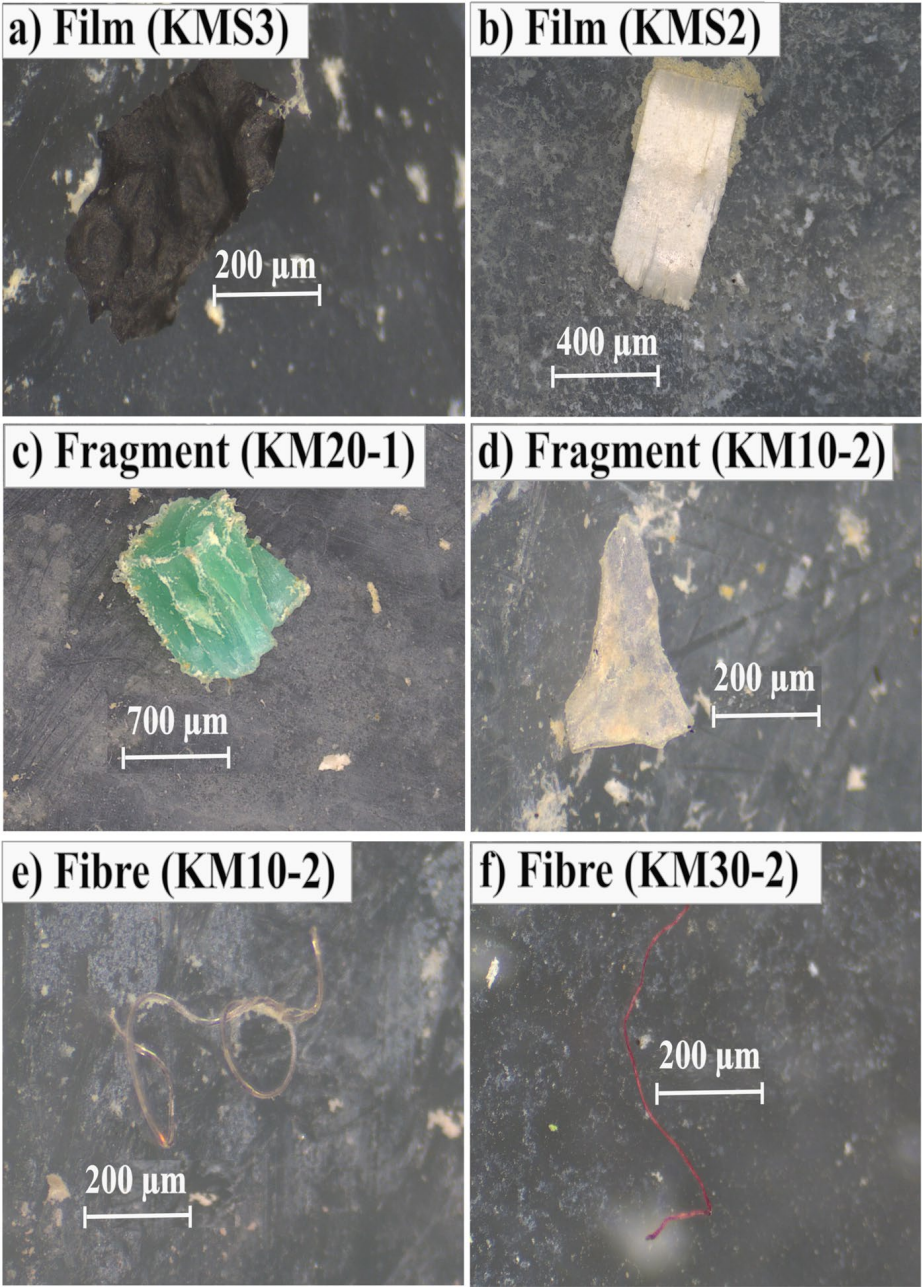
Representative images of microplastic (MP) categories isolated from agricultural soils in Kirimanjeshwara: **a**, **b** films, **c**, **d** fragments, and **e**, **f** fibres

Microplastics (MPs) retrieved from both surface and subsurface soil layers exhibited a diverse range of colours, with transparent MPs being the most dominant across all depths. Transparent particles accounted for 28.67% of all MPs in surface soils and 27.48% in subsurface soils (Supplementary Fig. S2). In surface samples, transparent MPs were followed by blue (24.83%), white (17.44%), black (13.61%), red (7.13%), green (4.76%), yellow (1.80%), pink (1.06%), orange (0.53%), and purple (0.13%). In subsurface samples, the relative proportions shifted slightly, with black MPs (22.48%) being the second most abundant, followed by white (19.50%), blue (14.88%), red (8.50%), green (4.03%), yellow (2.12%), brown (0.46%), pink (0.23%), orange (0.21%), and purple (0.09%) (Fig. 5).

### Polymer composition of microplastics

The ATR-FTIR analysis conducted on 100 individual MP particles revealed the presence of four distinct polymer types: polyethylene (PE), polypropylene (PP), polyester (PES) and polystyrene (PS). Among these, PE was the most abundant, accounting for 43.43% of the identified particles, followed by PP (35.55%), PS (8.99%), and PES (7.51%) (Supplementary Table S5; Supplementary Fig. S3). A small proportion (4.52%) of particles could not be identified due to low spectral match quality, as their Pearson correlation coefficient values were below the threshold of 0.8.

### Scanning electron microscopy–energy dispersive spectroscopy (SEM–EDS)

**Fig. 6:**
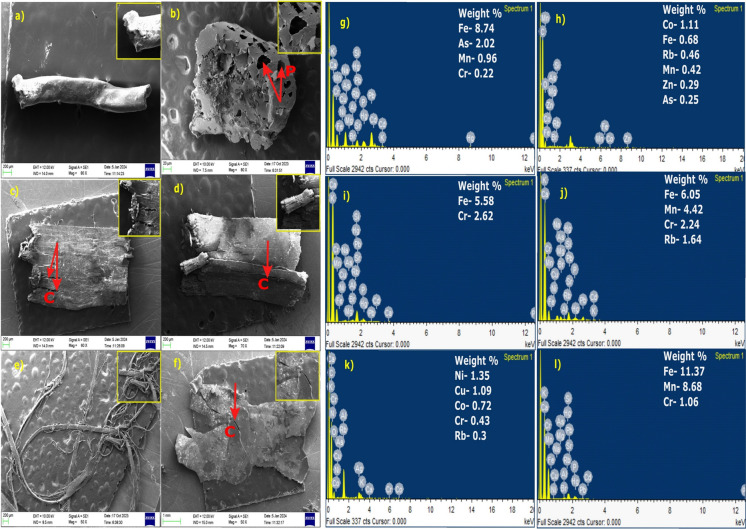
Scanning Electron Microscope (SEM) images of selected microplastic (MP) particles and their corresponding surface elemental compositions obtained through Energy Dispersive Spectroscopy (EDS). **a**, **b** SEM images of fragments with corresponding elemental concentrations shown in (**g**, **h**); **c**, **d**, **f** SEM images of films with corresponding elemental concentrations in (**i**, **j**, **l**); **e** SEM image of fibre with corresponding elemental concentrations in (**k**). The letters C and P in the figure denote cracks and pits, respectively, observed on the surfaces of the microplastic (MP) particles

To investigate the surface characteristics and elemental composition of MPs, selected particles were subjected to Scanning Electron Microscopy coupled with Energy Dispersive Spectroscopy (SEM–EDS). The SEM images revealed notable surface irregularities, including ridges, cracks, pits, and holes, indicative of weathering and mechanical degradation (Fig. 6). For instance, Fig. 6b clearly shows the presence of pits, while other MP morphologies exhibited pronounced fissures and surface fractures. The EDS analysis confirmed the presence of various metal elements adsorbed onto the surface of MPs. Detected metals included Fe, As, Mn, Cr, Co, Rb, Zn, Cu, and Ni.

### Risk assessment studies of microplastics

Microplastic (MP) pollution risk across the sampled sites was assessed using multiple indices, including the Pollution Hazard Index (PHI), Coefficient of Microplastic Impact (CMPI), Pollution Load Index (PLI), and Potential Ecological Risk Index (PERI).

The PHI values ranged from 19.97 to 123.50, with significantly higher values observed in mulched agricultural soils (average PHI: 98.62) compared to unmulched soils (average PHI: 53.89). The highest PHI (123.50) was recorded at KM3, while the lowest (19.97) was observed at KU3, an unmulched site (Supplementary Table S6). Based on hazard level classification, these values fall within Hazard Levels II–III, indicating moderate to high ecological risk. PHI was computed using polymer types such as polypropylene (PP), polyethylene (PE), polystyrene (PS), and polyester (PES) identified via ATR-FTIR spectroscopy.

The Coefficient of Microplastic Impact (CMPI) was applied to evaluate the potential environmental impact of MP morphologies:

Films exhibited an average CMPI of 0.55, placing them in the maximum impact category (0.51–0.80). The highest film-related CMPI (0.73) occurred at KM1, and the lowest (0.33) at KM3.

Fibres showed an average CMPI of 0.33, classifying them under the average impact category (0.11–0.50). The maximum CMPI for fibres (0.47) was found at KM2, while the lowest (0.17) was at KM1.

Fragments recorded an average CMPI of 0.12, also within the average impact category. The highest value (0.25) was noted at KM3, and the lowest (0.05) at KU2, an unmulched site (Supplementary Table S7).

PLI values ranged from 1.00 to 3.05, where values > 1 indicate pollution (Supplementary Table S8). All mulched sites (KMS1–KMS5) exhibited elevated PLI values between 2.27 and 3.05, suggesting moderate MP accumulation. In contrast, unmulched sites (KUS1–KUS3) displayed lower PLI values ranging from 1.00 to 1.61, with KUS2 at the baseline level (1.00). PERI values across the sites ranged from 38.49 to 237.36 (Supplementary Table S9).

Among mulched sites, PERI ranged from 157.32 (KMS5) to 237.36 (KMS3), placing them in the moderate ecological risk category (150–300). Unmulched sites displayed comparatively lower risk, with KUS3 (38.49) and KUS1 (116.41) categorized as low risk (< 150), and KUS2 (156.26) at the lower threshold of moderate risk.

These findings highlight the cumulative environmental and ecological burden of microplastics, particularly in mulched agricultural soils, underlining the need for targeted risk mitigation and regular monitoring.

### Risk assessment studies of trace elements

The Pollution Load Index (PLI) values for all sampled locations exceeded 1, indicating that the agricultural soils in the study area are polluted with respect to trace element concentrations (Supplementary Table S11).

The Geo-accumulation Index (I_geo_) values ranged from < 0 to < 2 across the sample locations, corresponding to I_geo_ classes 0 to 2. These values suggest contamination levels ranging from uncontaminated to moderately contaminated (Supplementary Table S12). Lead and Cr consistently exhibited I_geo_ values below 0 across all locations, indicating that these elements do not contribute to contamination. For Fe, I_geo_ values at KM4, KM5, KU1, and KU2 were between 0 and 1, suggesting slight to moderate contamination, while all other fields show values < 0, indicating no contamination. Manganese exhibited a similar pattern, with I_geo_ values in the uncontaminated to moderately contaminated range at most fields, except at KM1, KM2 and KU3, where values were < 0. Zinc showed more variability, with moderate contamination observed at KM1 (I_geo_  > 1), while KM5, KU1 and KU2 exhibited I_geo_ values < 0, suggesting no contamination. In the remaining fields, Zn values ranged between 0 and 1, reflecting slight to moderate contamination levels. These findings highlight spatial variability in trace element contamination, with moderate levels associated with specific fields.

The Potential Ecological Risk Index (PERI) values for all locations were below 150, placing the entire study area within the low ecological risk category (Supplementary Table S13). This suggests that despite localized contamination patterns, the overall ecological risk posed by trace elements in the agricultural soils of Kirimanjeshwara is minimal.

### Results of the principal component analysis

Principal Component Analysis (PCA) was conducted on standardized MP and trace element data from Kirimanjeshwara agricultural soils representing different cropping systems (mulched *vs.* unmulched) and soil depth. Bartlett’s test of sphericity confirmed the adequacy of the correlation matrix for factor analysis (χ^2^ = 452.24; df = 91; *p* < 0.001). The determinant of the correlation matrix (1.9856 × 10^−8^) indicated multicollinearity among variables. The Kaiser–Meyer–Olkin (KMO) which measures sampling adequacy, was found to be 0.6605, suggesting mediocre but suitable for performing the PCA.

**Fig. 7:**
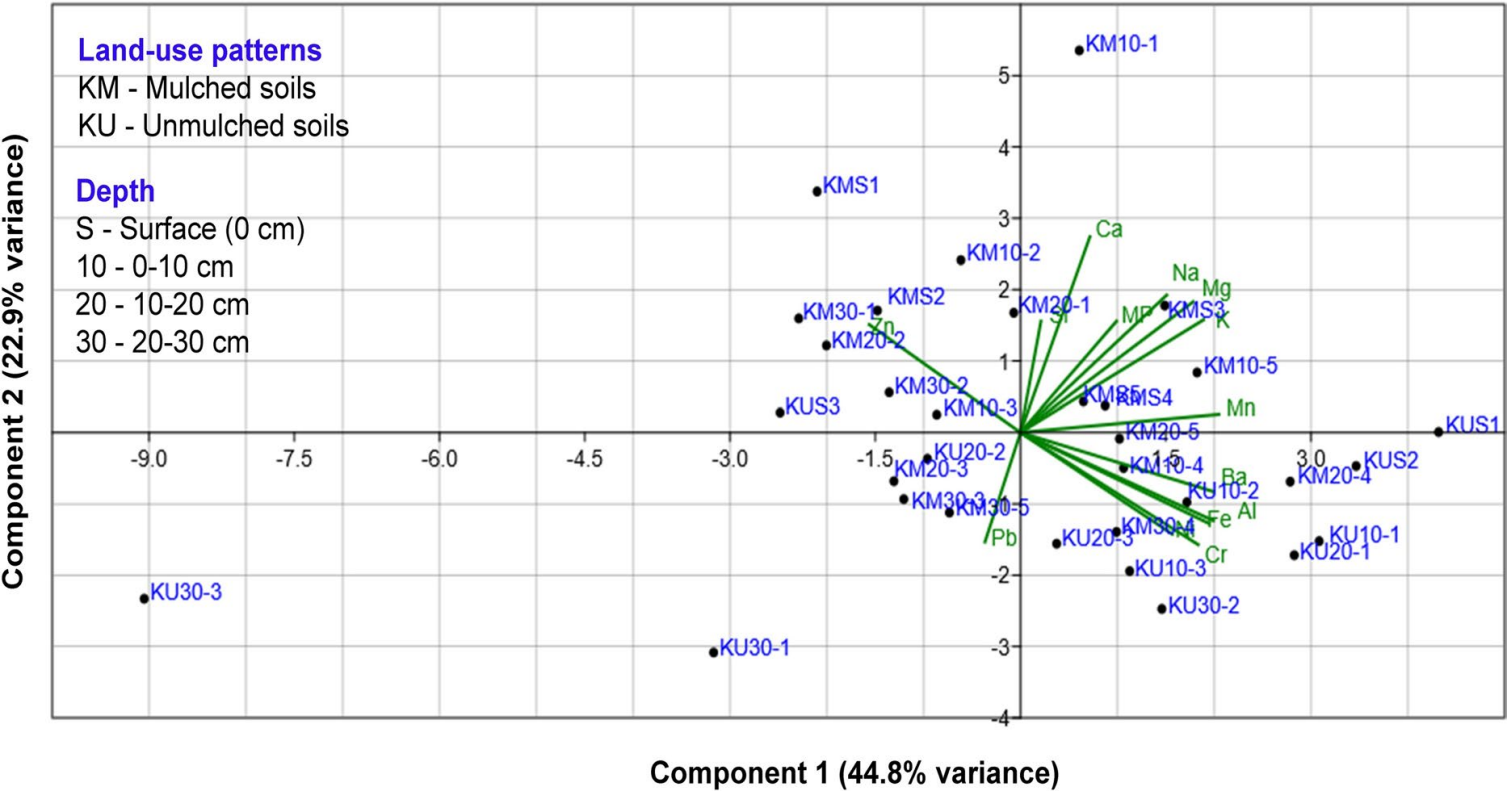
PCA biplot showing the relationship between soil samples and variables based on microplastic and trace elements data from mulched (KM) and unmulched (KU) fields in Kirimanjeshwara. PC1 and PC2 explain a combined 67.7% of the total variance

The first two principal components—PC1 and PC2—explained 44.8% and 22.9% of the total variance, respectively, accounting for a cumulative 67.7% of the overall variability in the dataset. The PCA score plot (Fig. 7) revealed a clear separation between mulched (KM) and unmulched (KU) soils. KM surface and subsurface samples clustered near the vectors of microplastics (MP), manganese (Mn), calcium (Ca), magnesium (Mg), and sodium (Na), indicating a strong association. In contrast, deeper KU samples—particularly those at 30 cm depth (e.g., KU30-3)—were positioned farther from MP-associated vectors, suggesting distinct geochemical profiles with minimal MP influence.

Interpretation of the PCA biplot was based on the proximity of sample scores to variable vectors, where closer alignment indicates stronger association. A depth-wise pattern was also evident: MP concentrations were highest in the surface and 10 cm layers and declined with depth, particularly in unmulched fields. Variable loadings showed strong alignment of MP with Mn, Ca, and Na, implying a coupled distribution pattern likely driven by mulching practices and surface-applied inputs.

## Discussion

### Abundance of microplastics

Among the surface soil samples, the highest MP abundance was observed at location KMS5 (490 items/kg), a mulched watermelon field, while the lowest was recorded at KUS2 (63.85 items/kg), an unmulched paddy field (Fig. 2a). This distinct contrast reinforces the hypothesis that mulched farmlands exhibit significantly higher MP contamination compared to unmulched farmlands. The widespread use of plastic mulch sheets in watermelon cultivation appears to be the primary contributor to elevated MP levels in these soils (Zhou et al., 2020). The findings are consistent with previous studies; for example, Huang et al., (2020) reported MP abundances of 308 ± 138.1 pieces/kg in soils under continuous mulching for 15 years. In the present study, mulching practices have been ongoing for over 15 years, which supports the similarity in observed MP concentrations. The persistent degradation of polyethylene mulch films likely leads to long-term accumulation of MPs in surface soils. In addition to mulching, other potential pathways of MPs in agricultural fields include irrigation water, surface runoff, atmospheric deposition, littering, and the application of polymer-based fertilizers (Machado et al., 2018). The lowest MP abundance in the current study was detected at KUS7, an unmulched paddy field, further emphasizing the influence of land management practices on MP distribution.

The notably high standard deviation in MP abundance at the 10–20 cm depth (Fig. 2b, 178.32 ± 184.08 items/kg) suggests considerable variability in MP distribution at this level. This depth-specific variability is particularly evident when considering the combined dataset from both mulched and unmulched samples. Such variation strongly implies that the 10–20 cm soil layer serves as a critical transition zone, where MP accumulation is heavily influenced by the downward migration and degradation of plastic mulch films applied at the surface. While the surface soil layer is subject to dynamic processes such as fresh fragmentation, wind erosion, and surface runoff, which may cause spatial heterogeneity, and the deeper 20–30 cm later often reflects dilution or attenuation through percolation and soil matric interactions, the intermediate 10–20 cm layer appears to retain a more distinguishable legacy of surface-applied plastic mulching. This is particularly evident in the pronounced difference in MP concentrations between mulched and unmulched samples at this depth, contributing to the elevated standard deviation across the dataset. These results underscore plastic mulching as a major source of subsurface MP contamination particularly in the upper subsoil and align with previous observations that prolonged mulching leads to vertical translocation of MPs into soil profiles (Zhou et al., 2020). Further investigation into MP transport dynamics, particularly in relation to tillage practices, bioturbation, and soil texture, is warranted to understand long-term accumulation trends in agricultural landscapes.

Microplastic abundance was found to be highest in the surface soil layer, progressively decreasing with depth, with the lowest concentrations observed at 20–30 cm. This depth-dependent trend aligns with findings from other studies that have reported similar patterns of declining MP concentrations with increasing soil depth (Zhang et al., 2022). The accumulation of MPs in surface soils is largely attributed to atmospheric deposition, limited vertical mobility, and bioturbation, which tends to concentrate particles near the surface. In contrast, deeper soil layers generally exhibit minimal MP presence unless disturbed by mechanical activities such as deep tillage or prolonged mulching (Qiu et al., 2023).

In the present study, mulched farmlands consistently showed a decline in MP concentration with increasing depth, reflecting typical plastic mulch degradation and limited vertical transport (Zhang et al., 2022). An exception was noted at KM1, where the middle layer (10–20 cm) exhibited higher MP abundance, potentially due to localized soil mixing or historical tillage activity. Unmulched paddy farmlands, on the other hand, showed greater variability in vertical MP distribution. While some fields followed the expected surface-dominant trend, others exhibited higher concentrations at subsurface depths, likely resulting from frequent ploughing and field preparation practices that facilitate deeper incorporation of MP particles.

### Characteristics of MPs

Small-sized MPs, particularly those less than 1 mm in diameter, were more prevalent across all agricultural fields studied. Among these, the 0.1–0.3 mm size fraction (Fig. 3a) was the most abundant. In contrasts, larger MPs (1–5 mm) were relatively scarce, especially in deeper soil layers (20–30 cm), where smaller MPs predominated (Fig. 3b). This distribution is likely influenced by particle mobility: smaller MPs can percolate more readily into subsurface horizons, while larger particles tend to remain near the surface due to their limited mobility and increased exposure to surface factors such as sunlight, oxidation, and physical abrasion (Miao et al., 2024).

Studies have revealed that mulched agricultural soils contain a higher proportion of small-sized MPs, particularly those below 0.5 mm, compared to unmulched soils (Ju et al., 2025). This is mainly due to the fragmentation of plastic mulch films, which degrade into smaller particles under environmental stressors such as ultraviolet radiation and mechanical disturbance. Liu et al., (2025) further reported that in mulched and fertilized fields exhibited approximately double the MP concentration of unmulched and unfertilized soils, emphasizing the role of intensive agricultural practices in MP generation.

In contrast, unmulched soils may contain a relatively higher proportion of medium-sized MPs (0.3–1 mm), potentially reflecting different contamination pathways and slower rates of fragmentation. These variations in size distribution across depth and land-use types underscore the complex and dynamic behaviour of MPs in soil environments. The observed patterns highlight the significance of mulching practices in promoting both the abundance and vertical distribution of smaller MPs, which may have implications for their persistence, transport, and ecological impacts within agricultural systems.

The vertical distribution of MPs was consistently observed across all surface and subsurface samples, regardless of land-use type. Similar morphological patterns were evident in both mulched and unmulched farmlands, with film-shaped MPs being the most dominant, followed by fibres and fragments. However, at greater depths—specifically in the 20–30 cm layer (Fig. 4b)—fibres became the predominant form, surpassing films and fragments. This shift suggests that the degradation and downward mobility of films diminish with depth, while fibres demonstrate a greater potential for vertical movement throughout the soil profile.

Soil properties play a pivotal role in shaping the vertical distribution of microplastics (MPs) and the behavior of associated trace elements. In the present study, the contrasting characteristics of lateritic and sandy soils likely influenced both MP retention and trace metal mobility. Lateritic soils, known for their higher clay content, elevated concentrations of iron and aluminum oxides, and relatively low permeability, are expected to restrict the vertical migration of MPs. These soils also offer greater sorption capacity for trace metals, owing to their high surface area and abundance of reactive mineral phases (Pang et al., 2023). In contrast, sandy soils—characterized by larger pore spaces, lower organic matter content, and reduced surface reactivity—are more conducive to deeper MP penetration and may facilitate the leaching of trace metals into subsoil layers (Gao et al., 2021). These intrinsic differences in soil texture and mineralogy likely account for the spatial variation observed in MP abundance and trace metal concentrations across the sampled sites.

The persistence of fibres at depth may be explained by their higher mobility and association with root systems, as demonstrated in previous studies. Tumwet et al., (2024) reported that microfibres, typically concentrated in the upper 0–20 cm, were also present in the 20–50 cm depth range, likely due to their attachment to plant roots and redistribution by root growth and soil disturbance. Similarly, Luo et al., (2024) documented that the fibrous morphology facilitates migration through soil pores, with fibres comprising 53.33% of surface MPs (0–3 cm) in Shanghai soils and 37.62% at 3–6 cm depth. These findings reinforce the notion that microfibres are more mobile and can be vertically translocated through physical and biological processes.

In contrast, the persistent presence of film-shaped MPs in surface layers, especially in mulched farmlands, is strongly linked to fragmentation of polyethylene (PE) mulch films. Long-term mulching practices contribute significantly to the deposition of PE films in agricultural soils (Li et al., 2022). This is supported by the present study, where film-shaped MPs were dominant in surface samples, and ATR-FTIR analysis confirmed PE as the primary polymer type. Additionally, the high proportion of black-coloured MPs—commonly associated with aged mulch films—further substantiates the connection between plastic mulch degradation and film MP accumulation. Together, these observations emphasize that morphological traits affect the vertical movement and persistence of MPs, with fibres showing higher mobility while films tend to remain on the surface due to slower degradation and a denser material structure. Grasping these dynamics is important for evaluating the persistent effects of plastic contaminants on soil ecosystems.

Transparent MPs were the most dominant across all soil compartments, likely originating from common consumer plastics such as bags, bottles, and synthetic fibres. The abundance of transparent MPs may also reflect progressive environmental weathering of coloured plastics. While physical abrasion in soil environments does not chemically bleach plastic, it can remove surface pigment layers, expose the less pigmented interior or accelerating pigment leaching (Rist et al., 2018). Over time, this results in coloured MPs transitioning into lighter or transparent particles, particularly under exposure to sunlight, oxidation, and microbial action.

Coloured MPs detected in the study—such as blue, red, green, yellow, black, and white—originated from diverse sources including household waste, agricultural films, and packaging materials. Black MPs, which were consistently observed in mulched soils, are typically associated with the degradation of black polyethylene mulch films, a common agricultural practice in the region. Meanwhile, the persistent presence of blue fibre-shaped MPs across depths, especially near roads, residential areas, and industrial zones, suggests a significant contribution from synthetic textile fibres. These findings align with Crossman et al. (2020), who reported that wastewater irrigation introduces substantial quantities of textile-derived microfibres—particularly blue fibres—into agricultural soils. In the present study area, irrigation primarily relies on open wells and surface water sources (ponds and canals), which are vulnerable to contamination from nearby anthropogenic activities, including untreated or poorly managed domestic and industrial effluents.

The colour-dependent degradation of MPs is also influenced by the type of pigments used. Black and white plastics, often pigmented with carbon black or titanium dioxide, exhibit greater resistance to UV degradation due to their ability to block solar radiation. In contrast, green, red, and yellow pigments, typically based on anthraquinone, phthalocyanine, or diketopyrrolopyrrole, offer limited UV protection and degrade more rapidly upon environmental exposure (Key et al., 2024). This explains the lower frequency of red, green, pink, yellow, and orange MPs in the study area. As pigments leach out or degrade, the residual MP particles are often reclassified as transparent during visual sorting, which contributes to their overrepresentation in the dataset. These findings demonstrate that colour distribution in soil MPs is shaped not only by source diversity but also by post-depositional transformations, including pigment stability, environmental exposure, and degradation kinetics. Understanding these transformations is essential for source tracking and for evaluating the behavior and longevity of MPs in terrestrial environments.

### Polymer composition of microplastic samples

Across both mulched and unmulched agricultural fields, polyethylene (PE) was the most prevalent polymer type identified, followed by polypropylene (PP). In mulched soils, the dominance of PE is largely attributed to the degradation of plastic mulch films, which are commonly composed of low-density polyethylene (Qi et al., 2020). In unmulched fields, however, the presence of PE and PP can be linked to other agricultural inputs such as seed bags, irrigation tubing, pesticide and fertilizer packaging, many of which are manufactured using these same polymers (Corradini et al., 2019; Zhou et al., 2023). Atmospheric deposition also contributes to the accumulation of these polymers in soil, particularly in areas exposed to road dust and anthropogenic emissions (Allen et al., 2019).

Although detected in lower concentrations, polystyrene (PS) was present in some samples and may originate from discarded food containers, seedling trays, or insulation materials used in field setups or irrigation infrastructure (Zhang et al., 2020a). The occurrence of polyester (PES) likely reflects contributions from ropes, clothing fibres, and fabric-based materials, which are commonly used in rural and peri-urban agricultural settings. PES may also be introduced via irrigation water, windborne fibres, or domestic activities, especially given the proximity of the study sites to roadways and residential zones (Zhang et al., 2020b).

The accumulation of these synthetic polymers in soil poses several ecological and agronomic risks. PE and PP residues can alter soil physical properties by reducing water retention, limiting aeration, and disrupting soil aggregation, which may impede root penetration and overall plant health. Additionally, polymer debris has been shown to affect soil microbial communities, invertebrate fauna, and biogeochemical cycles, particularly nitrogen transformation processes (Bläsing & Amelung, 2018; Huerta Lwanga et al., 2016). Moreover, MPs can adsorb environmental contaminants or leach plasticizers and stabilizers, leading to potential long-term risks for soil quality, crop safety, and human health (Boots et al., 2019). These results emphasize the necessity for monitoring polymer-specific contamination pathways and to inform the development of management strategies aimed at reducing the accumulation and effects of persistent plastic polymers in agricultural soils.

### SEM–EDS observations and microplastic-metal interactions

Scanning Electron Microscopy coupled with Energy Dispersive Spectroscopy (SEM–EDS) analysis showed distinct surface features on MP particles, including cracks, pits, and fissures, which are characteristic of environmental weathering and degradation (Dong et al., 2020). These morphological changes, resulting from prolonged exposure to physical, chemical, and biological processes in soil, increase the surface roughness and reactivity of MPs, making them effective sorption sites for various contaminants. Because of their high specific surface area, hydrophobic properties, and chemical stability, MPs are recognized as vectors for pollutants like fertilizers, pesticides, and trace elements (Prajapati et al., 2024; Qiu et al., 2022; Zhu et al., 2023).

Metals can be associated with MPs in two primary ways: they may be incorporated during manufacturing—as catalysts, pigments, or stabilizers (Liu et al., 2023; Pavlovskyi & Vorobyova, 2025)—or adsorbed from the surrounding environment. In this study, SEM–EDS spectra from selected MPs confirmed the presence of trace elements such as Mn, Fe, As, Cr, Cu, Ni, and Zn on particle surfaces (Fig. 5), suggesting post-depositional sorption. This adsorption is likely affected by soil pH, organic matter content, and redox conditions, which regulate the mobility and bioavailability of metals (Liu et al., 2024; Premarathna et al., 2023).

The detection of metal-laden MPs in agricultural soils highlights their role as reservoirs and carriers of environmental contaminants. Previous studies have shown that continuous inputs from fertilizers and pesticides can elevate the levels of Cu, Pb, and Zn found in the soil systems (Wang et al., 2024), increasing the likelihood of their attachment to MP surfaces. Once sorbed, MPs can facilitate the transport and redistribution of these metals, contributing to their persistence and ecological toxicity (An et al., 2023). This mechanism amplifies the environmental risk, as MPs not only introduce synthetic polymers into soils but also enhance the mobility of co-contaminants, potentially impacting plant uptake, microbial processes, and aquatic ecosystems (Pinisakul et al., 2023).

### Abundance of trace elements

The consistent distribution of trace elements across both surface and subsurface soils in the study area indicates the influence of both geogenic and anthropogenic factors. The high average concentrations of iron (Fe) can be primarily attributed to the region’s lateritic geology, where weathering of iron-rich parent rocks contributes to Fe enrichment in soils (Udayashankar & Sarvade, 2019). Lateritic soils, common in tropical regions like coastal Karnataka, are well known for their iron oxide-rich horizons, leading to naturally elevated Fe levels.

Other trace elements detected in the soils—zinc (Zn), chromium (Cr), manganese (Mn), and lead (Pb)—are commonly introduced through a combination of natural processes and agricultural practices. These include the application of chemical fertilizers, pesticides, manure, sewage sludge, and irrigation with contaminated surface water, as well as atmospheric deposition from nearby anthropogenic actions (An et al., 2023; Su et al., 2023). In the present study, Zn emerged as the second most abundant element, suggesting substantial input from both geogenic sources and agrochemicals, as Zn is frequently used in fertilizers and pesticide formulations.

The interaction between MPs and trace elements may further influence metal distribution, mobility, and bioavailability in soils. MPs act as sorptive surfaces for trace elements, potentially altering their geochemical behavior and facilitating redistribution through soil layers (Chakraborty et al., 2025). This highlights the importance of examining MP–metal interactions when evaluating pollution risks in agroecosystems.

In contrast, the relatively lower concentrations of Cr and Pb likely reflect the absence of major industrial sources, which are typically responsible for Cr contamination (Liang et al., 2021), and possibly lower natural background levels of these elements in the local lithology. Additionally, the limited mobility of Pb and Cr in soils—due to their tendency to form insoluble complexes or bind strongly to soil particles—may restrict their accumulation, especially in deeper layers (Elnajdi et al., 2023).

A comprehensive understanding of the genesis and transport mechanisms of trace elements in agricultural soils is important for evaluating ecological risks and ensuring food safety (Degvekar et al., 2025). Regional differences in geology, land use, and agricultural practices must be considered when interpreting metal abundance patterns and designing soil management strategies in similar agro environments.

### Risk assessment of microplastics

Microplastic contamination in agricultural soils can pose substantial ecological risks, not only due to their persistence but also through their interactions with soil microbial communities and physical processes. Experimental studies have shown that the addition of polyethylene (PE) and polypropylene (PP) MPs can significantly affect soil microbial enzyme activities and reduce their diversity in agricultural soils. These disruptions can affect nutrient cycling, organic matter decomposition, and overall soil health (Fei et al., 2020; Han et al., 2024).

The Coefficient of Microplastic Impact (CMPI) was employed in this study to assess the relative environmental risks associated with different MP shapes. Among the identified shapes, film-type MPs exhibited the highest average CMPI value (0.55), placing them in the “maximum impact” category (Supplementary Table S7). Their thin, flexible structure allows them to cover soil surfaces, potentially impeding gas exchange, water infiltration, and microbial activity (Lozano et al., 2024). In the mulched soils, where films are more prevalent, this could have long-term consequences for plant and soil health.

Fibres, with a moderate CMPI values of 0.33, are readily transported through soil pores and can be ingested by soil organisms. While they integrate more easily into the soil matrix and are less disruptive structurally, their ingestion by fauna can still pose ecotoxicological risks (Frost et al., 2022). In contrast, fragments—due to their irregular shape and lower mobility—had the lowest average CMPI value (0.12), placing them in the “average impact” category. These particles tend to produce localized effects and are less likely to interact extensively with soil organisms (Wiedner & Polifka, 2020). The results indicate that the shapes of MPs significantly influence the soil environment. Films, being flat and flexible, can cover the soil surface and disturb water and air flow, which may negatively affect soil microbes and plants. Fibres move more easily through the soil and may be eaten by soil organisms, but they are less likely to disturb soil structure. Fragments, which are irregular and less mobile, tend to stay in one place and have smaller, localized effects. Understanding how different shapes behave helps improve the accuracy of risk assessments and supports better planning to manage MP pollution in farming areas.

These findings underscore the need for targeted management strategies in areas with elevated microplastic (MP) loads—particularly mulched agricultural fields such as KM1 and KM2, where the Coefficient of Microplastic Impact (CMPI) values were highest. To mitigate MP accumulation, potential interventions could include the application of biochar, periodic soil remediation, or transitioning to biodegradable mulching films. Moreover, the consistent detection of hazardous polymer types in these soils emphasizes the urgency for regulatory oversight and the implementation of farmer awareness programs aimed at minimizing MP inputs and safeguarding long-term soil fertility and food security.

The Pollution Load Index (PLI) results further indicate that all sites except KUS2 are affected by MP contamination (PLI > 1) (Supplementary Table S8). Notably, mulched sites exhibited significantly higher PLI values than unmulched ones, reinforcing the conclusion that plastic mulching is a primary driver of MP accumulation in agricultural soils (Long et al., 2023). However, the presence of MPs even in unmulched areas suggests additional sources such as atmospheric deposition, irrigation water, or legacy contamination (Zhou et al., 2020).

The Potential Ecological Risk Index (PERI) assessment showed that mulched sites consistently exhibited moderate ecological risk, highlighting the cumulative environmental burden associated with prolonged or intensive plastic use. For instance, KMS3, with the highest PERI value (237.36), may reflect long-term mulch usage or poor post-harvest plastic management (Supplementary Table S9). Conversely, unmulched sites generally recorded lower PERI values, with KUS3 (38.49) representing the least impacted site. However, the moderate PERI observed at KUS2 (156.26) underscores that non-mulch sources—including irrigation, airborne deposition, and residual legacy pollution—also contribute to MP contamination. These patterns emphasize the significant influence of land management practices on the ecological risks posed by MPs in agroecosystems (Liu et al., 2025).

### Risk assessment of trace elements

The Pollution Load Index (PLI) values calculated for all agricultural soil samples indicate that the fields in Kirimanjeshwara are largely polluted with trace elements (Supplementary Table S11). This suggests that the combined concentrations of Cr, Fe, Mn, Pb and Zn may pose risks to soil quality and crop safety. The present findings resonate with the study by Ennaji et al., (2020), which reported that 63.33% of agricultural soils in their Moroccan study area exhibited similarly elevated PLI values. The elevated pollution levels in the current study are likely due to anthropogenic inputs such as contaminated irrigation water, chemical fertilizers, and pesticide applications—all of which are known contributors to trace element accumulation in agricultural soils (Bhatia et al., 2015).

The Geoaccumulation Index (I_geo_) results further reveal spatial variability in contamination levels. While most soil samples remain unpolluted, certain areas, particularly those with elevated zinc concentrations, show moderate pollution levels (Supplementary Table S12). This variation reflects differences in land management practices, local soil conditions, and exposure to agrochemical inputs.

Despite the pollution indicators observed in PLI and I_geo_, the Potential Ecological Risk Index (PERI) values across all fields are below the threshold of concern, indicating that the ecological risk posed by trace elements is currently low (Supplementary Table S13). Nevertheless, the cumulative presence of multiple trace elements and their potential interactions with other pollutants such as MPs necessitate ongoing monitoring and assessment. Sustainable land management and regular risk evaluations are essential to ensure long-term environmental health, maintain agricultural productivity, and detect any emerging threats in this ecologically sensitive coastal farming region.

### Multivariate assessment of MPs and trace elements

The PCA results highlight the impact of plastic mulch usage on surface soil geochemistry through coupled MP–metal interactions. In mulched fields (KM), high MP abundance co-occurred with elevated levels of Mn and major cations such as Ca, Na, and Mg, particularly in topsoil layers. This pattern suggests that degradation of polyethylene mulch films is a major source of MPs and may also contribute to the enrichment of these elements. Previous studies have similarly shown that long-term mulching increases MP accumulation in surface horizons (Miao et al., 2024). Some of the excess Ca and Mg could originate from mulch additives (e.g., calcium carbonate) or from agrochemicals trapped under the plastic cover. Microplastic fragments serve as effective sorptive surfaces for various contaminants because of their large surface area and hydrophobic properties. Laboratory and field evidence shows that MPs are capable of adsorbing trace elements, including Cu, Zn and Pb (Weber et al., 2022). In this study, the strong co-loading of Mn with MPs in KM soils suggests either surface binding or co-deposition of nutrients and contaminants with plastic debris.

In contrast, unmulched subsoils (e.g., KU30 samples) appeared spatially distinct in the PCA, indicating minimal MP influence at 30 cm depth. Without direct plastic input, these layers contain negligible MPs and show metal distributions shaped primarily by natural soil processes or limited vertical transport. This interpretation is supported by findings that MPs exhibit restricted downward movement in soils, with < 1% migrating below 8 cm even after 2 years (Schefer et al., 2025). Thus, in unmulched systems, deeper soil horizons remain largely unaffected by plastic contamination and associated metal interactions. Overall, the PCA findings demonstrate how agricultural mulching practices can restructure soil geochemistry by promoting the accumulation and redistribution of MPs and metals in surface layers. These results emphasize the need for integrated soil management strategies that consider not only nutrient and contaminant inputs as well as the chronic influence of plastic use on soil health and environmental sustainability.

## Environmental implications and limitations of this study

This study highlights the presence of MPs in terrestrial ecosystems, which is emerging as a significant soil pollutant. A critical concern is the interaction between MPs and metals**,** as MPs can act as vectors, modifying the bioavailability and toxicity of associated contaminants thereby increasing environmental risks. These combined impacts threaten overall soil health by disrupting its physical, chemical, and biological properties, thereby jeopardizing food security through impaired plant growth and the potential uptake of MPs into crops.

The presence of microplastics (MPs) and associated trace metals in agricultural soils raises critical concerns not only for ecosystem integrity but also for potential human exposure. MPs can be taken up by crops, either through root absorption of smaller particles or via surface adhesion, thereby entering the human food chain through consumption of contaminated produce. Additionally, the leaching of trace metals and nanoplastics into groundwater presents a significant risk to drinking water quality, especially in regions reliant on shallow aquifers.

Agricultural practices such as tillage, irrigation, and wind-driven soil erosion can further exacerbate the problem by resuspending MPs and metal-laden particles into the atmosphere, creating potential pathways for inhalation exposure via airborne dust. These diverse exposure routes underscore the necessity for integrated risk assessments and the adoption of precautionary management practices in agroecosystems where plastic residues are prevalent.

To safeguard food and water security, it is imperative to advance our understanding of the transport, transformation, and bioavailability of MPs and their co-contaminants across the soil–plant–human continuum. This knowledge is essential for developing mitigation strategies that address both ecological and human health risks associated with plastic pollution in agricultural landscapes.

Consequently, there is an urgent need for robust monitoring and management strategies, which include establishing standardized detection methods and comprehensive remediation approaches (Yang et al., 2021). The results also underscore the necessity of transitioning to sustainable alternatives to conventional plastics. This informs crucial policy implications that advocate for reduced plastic production, improved waste management, and the widespread adoption of eco-friendly materials. For the future, research should prioritize understanding the long-term ecological consequences of MPs, their interactions with soil organisms, and their pathways into the food web to develop truly effective mitigation strategies (Table 1).

**Table 1:** A summary of selected studies on MPs in agricultural soils worldwide

Location	MP concentration	Size	Shape	Instrumentation	Polymers	References
Guizhou, China	1150.60 ± 647.86 items/kg	10–100 μm	Fragments	Metallographic microscopy, SEM–EDS, and FT-IR	Polyethylene	Miao et al. (2024)
Xinjiang, China	12,589 pieces kg^−1^	0.02–0.1 mm, 0.1–0.5 mm, 0.5–1.0 mm and 1.0–5.0 mm	Films, beads and fibres	Stereomicroscope, SEM–EDS, and FT-IR	Polyethylene	Yu et al. (2023)
Haean Basin, Republic of Korea	356.8 particles kg^−1^	20–49, 50–99, 100–299, and 300 μm and above	Fragments, fibres	FTIR, μ-FTIR	polypropylene, polyethylene, polyethylene terephthalate, polyvinyl chloride, polyethylene amide, and polymethyl methacrylate	Chia et al. (2023)
Jingyang County, China	1955.00 n·kg^−1^	20–50 μm, 50–100 μm, 100–200 μm, and 200–500 μm	NA	LDIR	PE, PET, PU	Wang et al. (2024)
Kapurthala, Jandiala, and Jalandhar, Punjab	NA	100 μm to 1 mm	NA	Stereo microscoe, ATR-FTIR, XRD	polypropylene (PP), polystyrene (PS) polyethylene terephthalate (PET), polybutylene terepthalate (PBT), and polyethylene (PE)	Thakur et al. (2023)
Ernakulam, Kerala	45.6 ± 26.4 items kg⁻1	< 100 µm, 100 µm to 500 µm 500 µm and 1000 µm, and 1000 µm to 1500 µm	Fragments, Sheets, Filaments and pellets	Raman AFM- microscope, SEM	polypropylene, Low-density polyethylene, Polyester, and thermoplastic elastomer	Borah et al. (2024)
Coimbatore region, Tamilnadu	1650 items kg^−1^	< 500 µm, 500 to 1000 µm, 1000 to 2000 µm, and 2000 to 5000 µm	NA	Stereo microscope, ATR-FTIR	Polyethylene > Polyvinyl alcohol (PVA) > Polystyrene (PS)	Karthika et al. (2024)
Bhauri and Kokta region, Bhopal, Central India	307.5 ± 9.19 and 69.5 ± 4.95 particles	5–2 mm, 2–1 mm, and 1–0.5 mm	NA	Stereomicroscope, ATR-FTIR	polyethylene (PE), polypropylene (PP), polyvinyl chloride (PVC), polyethylene terephthalate (PET), and polystyrene (PS)	Singh et al. (2024)

Future research should prioritize the assessment of biodegradation potential of microplastics (MPs) under field conditions, with a focus on the role of native microbial communities, soil temperature, moisture, and other environmental drivers influencing degradation rates. In addition, there is a pressing need to investigate the phytotoxic effects of MPs and associated trace metals on crop germination, nutrient uptake, root development, and overall yield, as these could directly compromise food production and soil fertility. To mitigate long-term contamination, sustainable soil management strategies—such as biochar application, composting, and organic mulching—should be rigorously evaluated for their ability to immobilize, degrade, or reduce the mobility of MPs and trace metals. Such integrated approaches hold promise for restoring soil health and enhancing resilience in plastic-impacted agroecosystems.

Although SEM–EDS analysis provided useful insights into surface morphology and elemental presence on selected MP particles, the results were qualitative in nature. The quantification of metal load per MP particle was not conducted due to analytical limitations, including the absence of certified reference materials for such analyses. Future studies should adopt quantitative techniques, such as calibrated SEM–EDS, ICP-MS, or synchrotron-based elemental mapping, to accurately determine the metal sorption potential of MPs and better estimate ecotoxicological risks.

A key limitation of this study is the potential heterogeneity in initial soil conditions across mulched and unmulched plots. Variations in soil porosity, organic matter content, mineralogy, and adsorption capacity may have influenced the vertical distribution and retention of MPs and metals (Liu et al., 2024). To address this, future studies should employ paired or replicated plot designs with known pre-mulching baselines or longitudinal monitoring to better isolate the effects of plastic mulching. This study focused on MPs in the 0.1–5 mm range, with polymer identification limited to the 0.3–5 mm fraction due to instrumental resolution constraints of ATR-FTIR. Notably, the 0.1–0.3 mm fraction, which accounted for nearly 50% of the total MP abundance, remained uncharacterized. Additionally, sub-100 µm MPs, which are increasingly recognized for their ecological relevance, were not detected due to methodological limitations. Future investigations should incorporate advanced spectroscopic and thermal techniques such as μ-FTIR, Raman microspectroscopy, or pyrolysis–GC/MS to enable detection, quantification, and polymer-level characterization of smaller MPs in complex soil matrices.

## Conclusions

This study confirms the extensive presence of MPs in agricultural soils of Kirimanjeshwara, with surface concentrations reaching up to 1895.41 items/kg. The abundance of MP declined with depth, and mulched fields showed significantly higher levels (4715.7 items/kg) compared to unmulched fields (791.7 items/kg). The predominant size class was 0.1–0.3 mm, and MPs were primarily composed of films, fibres, and fragments, with transparent particles being the most common. The Combined Microplastic Pollution Index (CMPI) reflected maximum impact for films and moderate impact for fibres and fragments. Polyethylene was the most frequently identified polymer, followed by polypropylene, polystyrene, and polyester. The Polymer Hazard Index exceeded 1000 across all sites, classifying them under extreme risk. SEM–EDS analysis indicated the presence of various trace elements (Fe, As, Mn, Cr, Co, Rb, Zn, Cu, Ni) on MP surfaces, indicating their role as potential vectors for metal accumulation. Soil trace element concentrations followed the order Fe > Zn > Mn > Cr > Pb. While Pb was undetected in surface layers, it was present at depth. PLI values > 1 indicated trace element pollution, and I_geo_ values suggested conditions ranging from unpolluted to moderately contaminated. Despite this, the Potential Ecological Risk Index (PERI) remained below 150 at all sites, suggesting a low overall ecological risk.

PCA results provided further insight into the relationship between land use, MP distribution, and soil geochemistry. The first two components (PC1 and PC2) explained a cumulative 67.7% of the variance, with mulched soils clustering near variables such as MP, Mn, Ca, Na, and Mg, indicating strong associations with mulching practices. In contrast, deeper unmulched samples were positioned farther from MP-associated vectors, suggesting limited MP influence and more geogenically driven metal distributions. These multivariate patterns reinforce the observation that surface-applied plastic mulch significantly alters soil chemistry and contaminant dynamics. Overall, the findings highlight that mulching practices intensify MP accumulation and influence metal mobility in agricultural soils. Continued monitoring and detailed mechanistic research are necessary to achieve greater insight of the long-term ecological impacts of MP–metal interactions and their impacts on soil health and agricultural yields safety.

## Supplementary Information

Below is the link to the electronic supplementary material.

Supplementary file1 (DOCX 718 KB)

## Data Availability

Data is provided within the supplementary information file.
