# Supplementary material for: Microplastic and trace element contamination in coastal agricultural soils of southern India: a comparative risk assessment of mulched and unmulched fields
Source: Environ Geochem Health. 2025 Sep 14;47(10):436. doi: 10.1007/s10653-025-02746-9 (PMC12433925; doi:10.1007/s10653-025-02746-9)
Supplement: Supplementary file 1 — Supplementary file1 (DOCX 718 KB) [file 10653_2025_2746_MOESM1_ESM.docx]

**Spatial Variability and Risk Assessment of Microplastics and Heavy Metals in Mulched and Unmulched Agricultural Soils of Coastal Karnataka, India.**

Ashwathi C^a^, Anish Kumar Warrier^b *^ Monalisha Murmu^c^, Udita Priyadarsini^c^, and Santhosh Prabhu^a^

^a^Department of Civil Engineering, Manipal Institute of Technology, Manipal Academy of Higher Education, Manipal, India.

^b^Centre for Climate Studies, Department of Civil Engineering, Manipal Institute of Technology, Manipal Academy of Higher Education, Manipal 576104, India

^c^Department of Sciences, Manipal Institute of Technology, Manipal Academy of Higher Education, Manipal 576104, Karnataka, India.

^*^Corresponding author email address: [anish.warrier@manipal.edu](mailto:anish.warrier@manipal.edu)

**Supplementary File Table S1.** Abundance of MPs in surface samples

|  | **0.1-0.3 mm** | | | **0.3-1 mm** | | | **1-5 mm** | | |
| --- | --- | --- | --- | --- | --- | --- | --- | --- | --- |
| **Sample ID** | **FILM** | **FIBRE** | **FRAGMENT** | **FILM** | **FIBRE** | **FRAGMENT** | **FILM** | **FIBRE** | **FRAGMENT** |
| KMS1 | 50 | 10 | 15 | 55 | 5 | 4 | 2 | 0 | 0 |
| KMS2 | 8 | 14 | 2 | 1 | 13 | 4 | 3 | 0 | 4 |
| KMS3 | 25 | 26 | 16 | 32 | 7 | 7 | 5 | 0 | 41 |
| KMS4 | 34 | 6 | 6 | 27 | 10 | 7 | 8 | 1 | 2 |
| KMS5 | 58 | 38 | 9 | 44 | 32 | 8 | 7 | 0 | 0 |
| KUS1 | 29 | 6 | 5 | 10 | 0 | 1 | 3 | 0 | 1 |
| KUS2 | 6 | 1 | 0 | 12 | 4 | 0 | 1 | 0 | 0 |
| KUS3 | 10 | 3 | 2 | 9 | 3 | 1 | 3 | 0 | 0 |

**Supplementary File Table S2.** Abundance of MPs in Sub surface samples, (0-10cm, 10-20 cm and 20-30 cm)

|  | **0.1-0.3 mm** | | | **0.3-1 mm** | | | **1-5 mm** | | |
| --- | --- | --- | --- | --- | --- | --- | --- | --- | --- |
| **Sample ID** | **FILM** | **FIBRE** | **FRAGMENT** | **FILM** | **FIBRE** | **FRAGMENT** | **FILM** | **FIBRE** | **FRAGMENT** |
| KM10-1 | 36 | 1 | 2 | 42 | 10 | 6 | 7 | 1 | 0 |
| KM10-2 | 56 | 48 | 9 | 78 | 75 | 5 | 9 | 0 | 0 |
| KM10-3 | 7 | 18 | 3 | 10 | 18 | 1 | 6 | 0 | 0 |
| KM10-4 | 37 | 27 | 15 | 18 | 23 | 5 | 7 | 0 | 1 |
| KM10-5 | 7 | 12 | 3 | 12 | 12 | 3 | 0 | 0 | 0 |
| KU10-1 | 7 | 9 | 2 | 16 | 5 | 4 | 1 | 0 | 1 |
| KU10-2 | 3 | 0 | 0 | 3 | 0 | 2 | 0 | 0 | 0 |
| KU10-3 | 5 | 4 | 1 | 7 | 10 | 0 | 0 | 0 | 0 |
| KM20-1 | 99 | 22 | 13 | 57 | 21 | 6 | 4 | 0 | 1 |
| KM20-2 | 19 | 34 | 8 | 36 | 21 | 4 | 1 | 0 | 0 |
| KM20-3 | 9 | 22 | 0 | 0 | 20 | 2 | 0 | 0 | 1 |
| KM20-4 | 36 | 15 | 6 | 7 | 23 | 4 | 1 | 0 | 0 |
| KM20-5 | 3 | 0 | 4 | 5 | 7 | 2 | 0 | 0 | 0 |
| KU20-1 | 3 | 1 | 0 | 6 | 3 | 2 | 0 | 0 | 0 |
| KU20-2 | 3 | 1 | 0 | 1 | 0 | 0 | 0 | 0 | 0 |
| KU20-3 | 8 | 0 | 2 | 11 | 0 | 4 | 1 | 0 | 0 |
| KM30-1 | 6 | 15 | 0 | 8 | 1 | 1 | 0 | 0 | 0 |
| KM30-2 | 1 | 6 | 0 | 0 | 9 | 0 | 0 | 0 | 0 |
| KM30-3 | 0 | 2 | 0 | 0 | 4 | 0 | 0 | 0 | 0 |
| KM30-4 | 5 | 4 | 0 | 4 | 3 | 0 | 0 | 0 | 1 |
| KM30-5 | 4 | 7 | 1 | 6 | 1 | 0 | 0 | 0 | 0 |
| KU30-1 | 2 | 9 | 0 | 13 | 3 | 0 | 0 | 0 | 0 |
| KU30-2 | 2 | 11 | 0 | 2 | 6 | 1 | 0 | 0 | 0 |
| KU30-3 | 1 | 0 | 0 | 5 | 0 | 0 | 0 | 0 | 0 |

**Supplementary File Table S3.**  The heavy metal concentration of surface samples

| SampleList | Cr | Fe | Mn | Pb | Zn |
| --- | --- | --- | --- | --- | --- |
| KMS1 | 31.42 | 4507.57 | 74.12 | 0 | 558.55 |
| KMS2 | 42.15 | 5183.2 | 76.76 | 0 | 421.58 |
| KMS3 | 68.52 | 10010.5 | 118.86 | 0 | 297.70 |
| KMS4 | 72.95 | 9433.35 | 110.89 | 0 | 286.38 |
| KMS5 | 74.54 | 9711.98 | 109.29 | 0 | 173.34 |
| KUS1 | 93.05 | 16125.99 | 108.89 | 0 | 207.95 |
| KUS2 | 89.80 | 13430.14 | 108.17 | 0 | 162.28 |
| KUS3 | 48.47 | 5163.57 | 74.64 | 0 | 172.07 |

**Supplementary File Table S4.**  The heavy metal concentration of sub surface samples

| SampleList | Cr | Fe | Mn | Pb | Zn |
| --- | --- | --- | --- | --- | --- |
| KM10-1 | 45.35 | 6442.72 | 89.71 | 42.52 | 544.09 |
| KM10-2 | 45.07 | 5948.10 | 86.25 | 31.21 | 545.83 |
| KM10-3 | 62.19 | 8411.42 | 89.52 | 51.38 | 291.26 |
| KM10-4 | 65.70 | 8694.76 | 116.76 | 54.59 | 338.47 |
| KM10-5 | 86.81 | 8349.59 | 118.15 | 38.71 | 162.44 |
| KU10-1 | 99.10 | 14866.40 | 104.80 | 66.12 | 161.82 |
| KU10-2 | 81.24 | 10808.32 | 98.78 | 47.22 | 182.27 |
| KU10-3 | 127.73 | 8700.46 | 91.57 | 56.22 | 201.37 |
| KM20-1 | 41.11 | 6593.50 | 86.95 | 47.73 | 445.39 |
| KM20-2 | 45.79 | 6356.12 | 76.64 | 68.18 | 464.63 |
| KM20-3 | 63.15 | 6480.37 | 87.52 | 55.28 | 277.20 |
| KM20-4 | 86.65 | 11390.68 | 129.39 | 62.12 | 284.63 |
| KM20-5 | 80.03 | 11318.38 | 116.98 | 49.29 | 194.96 |
| KU20-1 | 94.24 | 13022.86 | 106.62 | 60.86 | 162.95 |
| KU20-2 | 70.95 | 6805.66 | 93.07 | 49.21 | 185.15 |
| KU20-3 | 74.97 | 8670.44 | 80.23 | 45.64 | 168.02 |
| KM30-1 | 42.11 | 5448.09 | 74.09 | 47.91 | 532.00 |
| KM30-2 | 51.90 | 9660.11 | 67.40 | 58.19 | 501.60 |
| KM30-3 | 61.67 | 7753.46 | 78.85 | 53.94 | 292.00 |
| KM30-4 | 72.97 | 11554.46 | 82.00 | 55.92 | 300.34 |
| KM30-5 | 87.20 | 8574.43 | 81.86 | 68.66 | 140.20 |
| KU30-1 | 67.55 | 7864.41 | 85.54 | 67.94 | 186.85 |
| KU30-2 | 108.90 | 15467.75 | 88.82 | 64.33 | 133.01 |
| KU30-3 | 0 | 2103.26 | 0 | 58.28 | 874.18 |

**Supplementary File Table S5.** Types of polymers present in each field. (Combined data from surface and subsurface samples in each field)

| Sample location | PE (Quantity) | PE % | PP (Quantity) | PP % | PS (Quantity) | PS % | PES (Quantity) | PES % | Others (Quantity) | Others % |
| --- | --- | --- | --- | --- | --- | --- | --- | --- | --- | --- |
| KM1 | 228 | 45.6 | 184 | 36.8 | 37 | 7.4 | 35 | 7 | 16 | 3.2 |
| KM2 | 176 | 37.61 | 162 | 34.62 | 67 | 14.32 | 44 | 9.4 | 19 | 4.06 |
| KM3 | 115 | 40.78 | 89 | 31.56 | 36 | 12.77 | 29 | 10.28 | 13 | 4.61 |
| KM4 | 158 | 46.04 | 112 | 32.65 | 31 | 9.04 | 24 | 7 | 18 | 5.25 |
| KM5 | 137 | 48.07 | 102 | 35.79 | 16 | 5.61 | 19 | 6.67 | 11 | 3.86 |
| KU1 | 52 | 36.62 | 72 | 50.7 | 4 | 2.82 | 7 | 4.93 | 7 | 4.93 |
| KU2 | 22 | 37.29 | 24 | 40.68 | 2 | 3.39 | 4 | 6.78 | 7 | 11.86 |
| KU3 | 54 | 60 | 26 | 28.89 | 2 | 2.22 | 1 | 1.11 | 7 | 7.78 |
| **Total** | **942** | **43.43** | **771** | **35.55** | **195** | **8.99** | **163** | **7.51** | **98** | **4.52** |

**Supplementary File Table S6.**  The PHI values of each field

| Sample Location | PHI |
| --- | --- |
| KM1 | 85.794 |
| KM2 | 113.795 |
| KM3 | 123.50 |
| KM4 | 86.2624 |
| KM5 | 81.7965 |
| KU1 | 60.4437 |
| KU2 | 81.2542 |
| KU3 | 19.9667 |

**Supplementary File Table S7.**  The CMPI values of each field

| Sample location | CMPI (FILM) | CMPI (FIBRE) | CMPI (FRAGMENT) |
| --- | --- | --- | --- |
| KM1 | 0.73 | 0.17 | 0.1 |
| KM2 | 0.45 | 0.47 | 0.08 |
| KM3 | 0.33 | 0.41 | 0.25 |
| KM4 | 0.54 | 0.33 | 0.14 |
| KM5 | 0.51 | 0.38 | 0.11 |
| KU1 | 0.63 | 0.25 | 0.11 |
| KU2 | 0.56 | 0.39 | 0.05 |
| KU3 | 0.66 | 0.22 | 0.11 |
| **Total** | **0.55** | **0.33** | **0.12** |

**Supplementary File Table S8.**  The PLI (MPs) values of each field

| Sample Location | PLI | PLI zone |
| --- | --- | --- |
| KMS1 | 3.05 | 2.99 |
| KMS2 | 2.94 |  |
| KMS3 | 2.27 |  |
| KMS4 | 2.5 |  |
| KMS5 | 2.3 |  |
| KUS1 | 1.61 |  |
| KUS2 | 1 |  |
| KUS3 | 1.26 |  |

**Supplementary File Table S9.**  The PERI (MPs) values of each field

| Sample Location | PERI |
| --- | --- |
| KMS1 | 165.03 |
| KMS2 | 218.72 |
| KMS3 | 237.36 |
| KMS4 | 165.89 |
| KMS5 | 157.32 |
| KUS1 | 116.41 |
| KUS2 | 156.26 |
| KUS3 | 38.49 |

**Supplementary File Table S10.** Overall concentrations of trace metals for each field, with background values highlighted in bold.

| SampleList | Cr | Fe | Mn | Pb | Zn |
| --- | --- | --- | --- | --- | --- |
| KMS1 | **40** | **5747.97** | 81.2169 | **34.54** | 520 |
| KMS2 | 46.23 | 6786.88 | 76.76 | 39.39 | 483.41 |
| KMS3 | 63.88 | 8163.94 | 93.69 | 40.15 | 289.54 |
| KMS4 | 74.57 | 10268.31 | 109.76 | 43.16 | 302.46 |
| KMS5 | 82.14 | 9488.6 | 106.57 | 39.16 | 167.73 |
| KUS1 | 88.48 | 12969.92 | 101.46 | 48.73 | 179.89 |
| KUS2 | 87.72 | 11627.97 | 97.21 | 40.19 | **165.68** |
| KUS3 | 62.79 | 6159.43 | **61.61** | 40.03 | 353.91 |

**Supplementary File Table S11.**  The PLI value of each field

| Sample List | PLI |
| --- | --- |
| KMS1 | 1.21 |
| KMS2 | 1.29 |
| KMS3 | 1.35 |
| KMS4 | 1.54 |
| KMS5 | 1.34 |
| KUS1 | 1.52 |
| KUS2 | 1.39 |
| KUS3 | 1.21 |

**Supplementary File Table S12.**  The I_geo_ value for individual heavy metal in each field

|  | Cr (I_geo_) | Fe (I_geo_) | Mn (I_geo_) | Pb (I_geo_) | Zn (I_geo_) |
| --- | --- | --- | --- | --- | --- |
| KMS1 | -1.24 | -0.58 | -0.19 | -0.58 | 1.06 |
| KMS2 | -1.03 | -0.34 | -0.27 | -0.39 | 0.96 |
| KMS3 | -0.56 | -0.08 | 0.02 | -0.37 | 0.22 |
| KMS4 | -0.34 | 0.25 | 0.25 | -0.26 | 0.28 |
| KMS5 | -0.20 | 0.14 | 0.21 | -0.40 | -0.57 |
| KUS1 | -0.09 | 0.59 | 0.13 | -0.08 | -0.47 |
| KUS2 | -0.10 | 0.43 | 0.07 | -0.37 | -0.58 |
| KUS3 | -0.58 | -0.48 | -0.58 | -0.37 | 0.51 |

**Supplementary File Table S13.**  The PERI value for each heavy metal in each field

| SampleList | Cr (EF) | Mn(EF) | Pb(EF) | Zn(EF) | PERI |
| --- | --- | --- | --- | --- | --- |
| KMS1 | 1.27 | 1.32 | 5.00 | 3.14 | 10.73 |
| KMS2 | 1.47 | 1.25 | 5.70 | 2.92 | 11.34 |
| KMS3 | 2.03 | 1.52 | 5.81 | 1.75 | 11.12 |
| KMS4 | 2.37 | 1.78 | 6.25 | 1.83 | 12.23 |
| KMS5 | 2.62 | 1.73 | 5.67 | 1.01 | 11.03 |
| KUS1 | 2.82 | 1.65 | 7.05 | 1.09 | 12.60 |
| KUS2 | 2.79 | 1.58 | 5.82 | 1 | 11.19 |
| KUS3 | 2 | 1 | 5.80 | 2.14 | 10.93 |


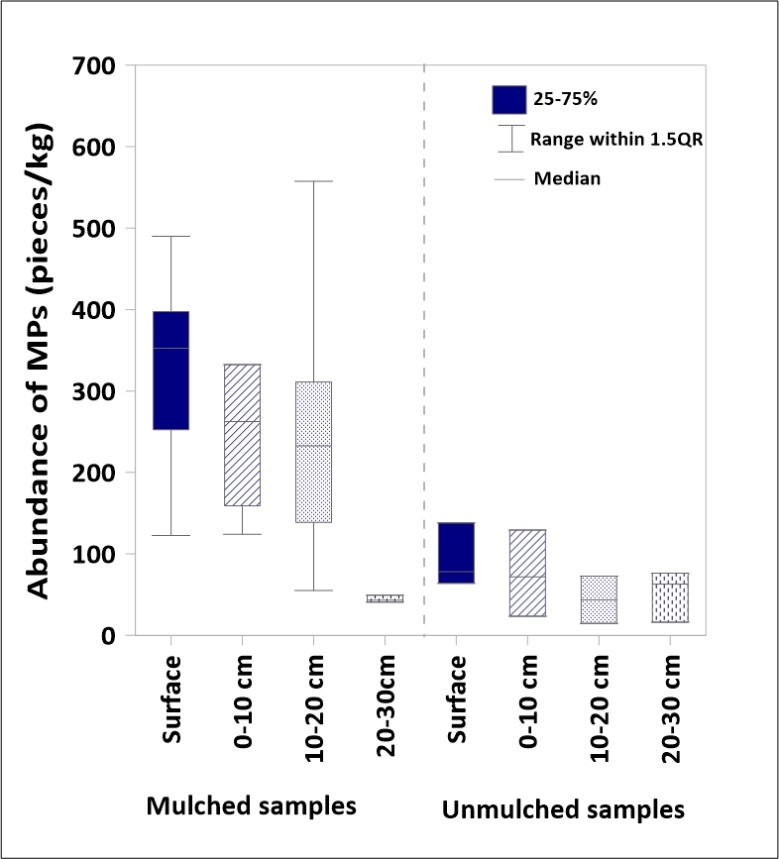


**Supplementary Fig. 1** Box plot for the mulched and unmulched samples in the surface and sub-surface samples


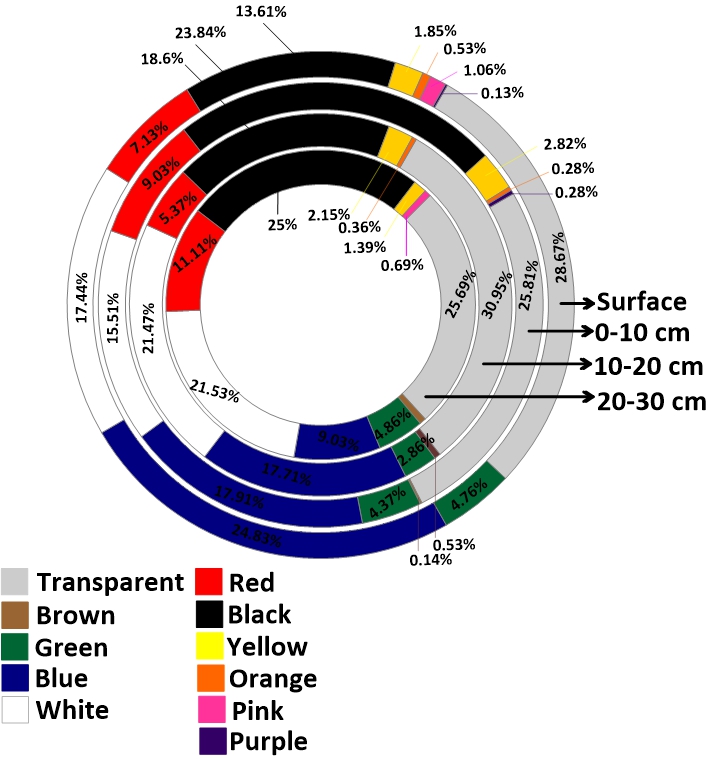


**Supplementary Fig. 2** The donut chart showing the colours of MPs in surface and sub-surface samples


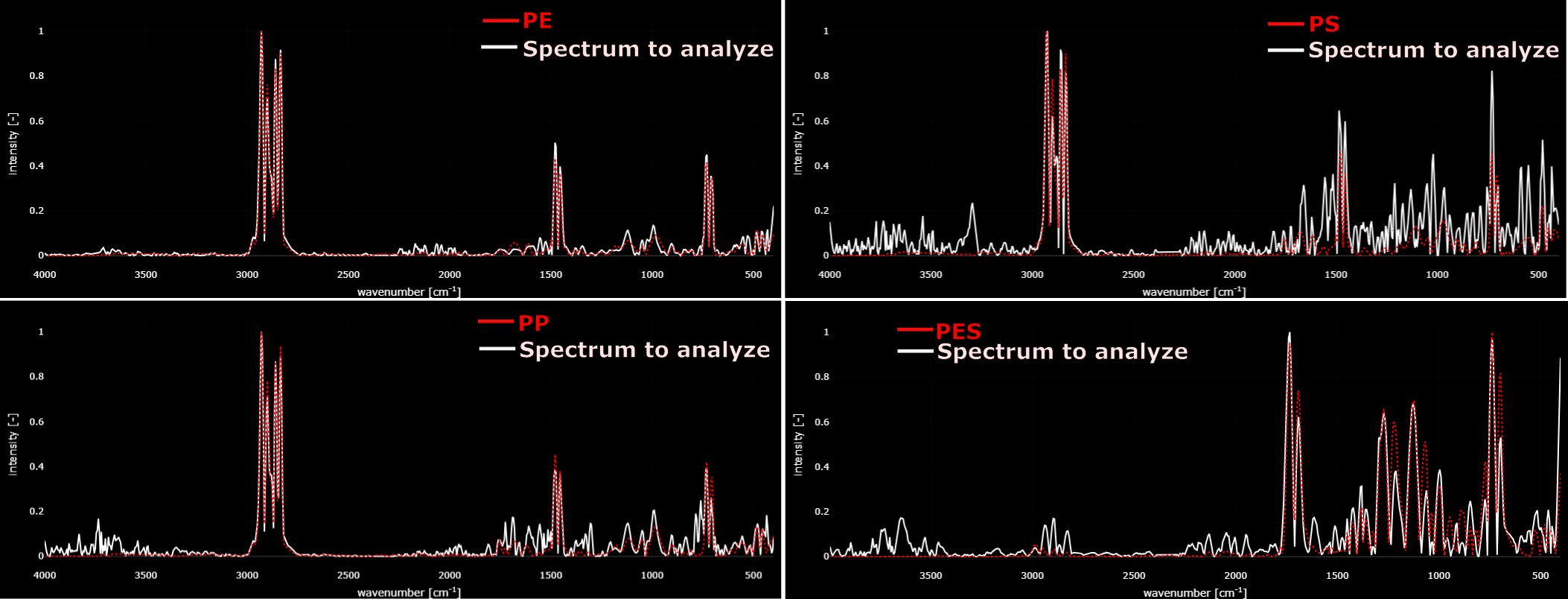


**Supplementary Fig. 3** IR Spectra of identified polymers.
